# Mitochondrial and nuclear DNA reveals reticulate evolution in hares (*Lepus* spp., Lagomorpha, Mammalia) from Ethiopia

**DOI:** 10.1371/journal.pone.0180137

**Published:** 2017-08-02

**Authors:** Zelalem Tolesa, Endashaw Bekele, Kassahun Tesfaye, Hichem Ben Slimen, Juan Valqui, Abebe Getahun, Günther B. Hartl, Franz Suchentrunk

**Affiliations:** 1 Department of Biology, Hawassa University, Hawassa, Ethiopia; 2 Zoologisches Institut, Christian-Albrechts-Universität zu Kiel, Kiel, Germany; 3 Research Institute of Wildlife Ecology, University of Veterinary Medicine Vienna, Vienna, Austria; 4 Department of Microbial, Cellular, and Molecular Biology, Addis Ababa University, Addis Ababa, Ethiopia; 5 Centre of Biotechnology, Addis Ababa University, Addis Ababa, Ethiopia; 6 Institut Supérieur de Biotechnologie de Béja, Avenue Habib Bourguiba, Béja, Tunisia; 7 Department of Zoological Sciences, Addis Ababa University, Addis Ababa, Ethiopia; National Cheng Kung University, TAIWAN

## Abstract

For hares (*Lepus* spp., Leporidae, Lagomorpha, Mammalia) from Ethiopia no conclusive molecular phylogenetic data are available. To provide a first molecular phylogenetic model for the Abyssinian Hare (*Lepus habessinicus*), the Ethiopian Hare (*L*. *fagani*), and the Ethiopian Highland Hare (*L*. *starcki*) and their evolutionary relationships to hares from Africa, Eurasia, and North America, we phylogenetically analysed mitochondrial ATPase subunit 6 (ATP6; n = 153 / 416bp) and nuclear transferrin (TF; n = 155 / 434bp) sequences of phenotypically determined individuals. For the hares from Ethiopia, genotype composition at twelve microsatellite loci (n = 107) was used to explore both interspecific gene pool separation and levels of current hybridization, as has been observed in some other *Lepus* species. For phylogenetic analyses ATP6 and TF sequences of *Lepus* species from South and North Africa (*L*. *capensis*, *L*. *saxatilis*), the Anatolian peninsula and Europe (*L*. *europaeus*, *L*. *timidus*) were also produced and additional TF sequences of 18 *Lepus* species retrieved from GenBank were included as well. Median joining networks, neighbour joining, maximum likelihood analyses, as well as Bayesian inference resulted in similar models of evolution of the three species from Ethiopia for the ATP6 and TF sequences, respectively. The Ethiopian species are, however, not monophyletic, with signatures of contemporary uni- and bidirectional mitochondrial introgression and/ or shared ancestral polymorphism. *Lepus habessinicus* carries mtDNA distinct from South African *L*. *capensis* and North African *L*. *capensis* sensu lato; that finding is not in line with earlier suggestions of its conspecificity with *L*. *capensis*. *Lepus starcki* has mtDNA distinct from *L*. *capensis* and *L*. *europaeus*, which is not in line with earlier suggestions to include it either in *L*. *capensis* or *L*. *europaeus*. *Lepus fagani* shares mitochondrial haplotypes with the other two species from Ethiopia, despite its distinct phenotypic and microsatellite differences; moreover, it is not represented by a species-specific mitochondrial haplogroup, suggesting considerable mitochondrial capture by the other species from Ethiopia or species from other parts of Africa. Both mitochondrial and nuclear sequences indicate close phylogenetic relationships among all three *Lepus* species from Ethiopia, with *L*. *fagani* being surprisingly tightly connected to *L*. *habessinicus*. TF sequences suggest close evolutionary relationships between the three Ethiopian species and Cape hares from South and North Africa; they further suggest that hares from Ethiopia hold a position ancestral to many Eurasian and North American species.

## Introduction

Hares and jackrabbits (genus *Lepus*; Leporidae; order Lagomorpha; Mammalia) are a difficult group, systematically, due to their large intraspecific phenotypic variation, their wide phenotypic overlap among species (e.g., Angermann[1,2], Palacios[3,4], Flux and Angermann[5]; see also Suchentrunk et al. [6]), and their shallow evolutionary divergence with the possibility of reticulate evolution (e.g., Thulin et al.[7], Alves et al.[8], Liu et al.[9], Melo-Ferreira et al.[10,11]). Traditional taxonomy based on phenotypic and morphological characters has earlier led to the description of a bewildering number of forms, often given species or even genus ranks. Many earlier species descriptions, however, were based on limited numbers of specimens, characters with doubtful systematic meaning, or limited geographical sampling. This has led to many synonyms or unsecure taxa that are currently classified into around 30 species, depending on the underlying species concept, available chorological, phenotypic, morphological, and molecular data (e.g., Flux and Angermann[5], Hoffmann and Smith[12], Alves and Hackländer[13]). Nevertheless, the systematic status of a fair number of forms is still uncertain; several may prove conspecific or separate species, once meaningful and representative morphological, geographical, and molecular data are available.

Starting with Thulin et al.[7], several molecular studies have identified patterns of reticulate evolution and paraphyly in a number of *Lepus* species, both on the intraspecific (e.g., Kasapidis et al.[14]; Stamatis et al.[15]) and interspecific levels (e.g., Alves et al.[8,16], Thulin et al.[17], Melo-Ferreira et al.[18], Wu et al.[19], Fredsted et al.[20], Ben Slimen et al.[21]; Melo-Ferreira et al.[22], Alves et al.[23,24], Pietri et al.[25], Zachos et al.[26], Liu et al.[9], Wu et al.[27], Melo-Ferreira et al.[28], Melo-Ferreira et al.[10,11], Mengoni et al.[29]). This may add to systematic ambiguity or confusion, particularly if phylogenetic conclusions are based solely on mtDNA data (e.g., Waltari and Cook[30]; Wu et al.[19], Ben Slimen et al.[31]) or geographically limited sample sizes, or when molecular samples are analyzed without concomitant phenotypic examination.

Historic and ongoing introgressive hybridization in natural populations upon secondary contact together with shared ancestral polymorphism due to incomplete lineage sorting do complicate evolutionary and systematic inference of the genus *Lepus*. Moreover, phenotypic or morphological characters and molecular markers may or may not concordantly indicate evolutionary divergence between subspecies or species (e.g., Bonhomme, et al.[32] Palacios[3], Alves et al.[8,16], Melo-Ferreira et al.[10], for hares from the Iberian Peninsula; Palacios[4], Pierpaoli et al.[33], Riga et al.[34], for *L*. *corsicanus* and *L*. *europaeus*; Yom-Tov[35], Suchentrunk et al.[36], Ben Slimen et al.[37], for hares from Israel; Ben Slimen et al.[38,21], for hares from Tunisia; Palacios et al.[39], and Suchentrunk et al.[40,41], for cape hares from South Africa; Scandura et al.[42] and Canu et al.[43] for hares from Sardinia; Liu et al.[9,44] for hares from China; Nunome et al.[45], for Japanese hares).

For Africa, six species of the genus *Lepus* are currently provisionally acknowledged: the cape hare, *Lepus capensis* L., 1758; the scrub hare, *L*. *saxatilis*, F. Cuvier, 1823; the African savanna hare, *L*. *victoriae* Thomas, 1893 (the currently often used name “*L*. *microtis* Heuglin, 1865” is considered a “*nomen dubium*”, following R. Angermann, unpubl., IUCN Lagomorph Specialist Group meeting 2012, Vienna, Austria, and should not be used; see also Petter[46], Angermann and Feiler[47], Suchentrunk et al.[6]); the Abyssinian hare, *L*. *habessinicus*, Hemprich and Ehrenberg, 1832; the Ethiopian hare, *L*. *fagani* Thomas, 1902; and the Ethiopian highland hare, *L*. *starcki* Petter, 1963 [5,12, 48,13,49]. Whereas *L*. *capensis* and *L*. *victoriae* are traditionally considered to occur across large parts of Africa including East Africa (e.g., Flux and Angermann[5]; Alves and Hackländer[13]), *L*. *saxatilis* occurs only in southern Africa (but see e.g., Collins[50], who includes *L*. *victoriae* in *L*. *saxatilis*), *L*. *fagani* and *L*. *starcki* have so far been described only from Ethiopia, and *L*. *habessinicus* from Eritrea, Ethiopia, and Somalia. The presence of *L*. *habessinicus* or *L*. *fagani* in other regions of East Africa (i.e., Djibouti, Sudan, South Sudan, Kenya, Uganda) is likely or possible [12, 48, 49]. *Lepus habessinicus* may be conspecific with *L*. *capensis* Angermann[1,2], *L*. *victoriae* may be conspecific with *L*. *saxatilis* (e.g., Robinson and Dippenaar[51]), and *L*. *capensis*-type forms from north of southern Africa (i.e., *L*. *capensis* sensu lato) may belong to one or more different species (e.g., Hoffmann and Smith[12], Smith and Johnston[48]). However, no meaningful molecular data exist for conclusive systematic decisions. As regards *L*. *capensis* sensu lato, molecular data so far are equivocal for the hypothesis of conspecificity of populations from South and North Africa[38, 52, 21, 31, 37,6].

For hares from East Africa published molecular phylogenetic data are only available for two mtDNA sequences, one from *L*. *habessinicus* and one from *L*. *starcki* Pierpaoli et al.[33]; their very close phylogenetic relationship suggests either shared ancestral polymorphism or recent introgression, conspecificity, incorrect phenotypical species identification, or a mix-up of samples. In this study we analyze for the first time evolutionary relationships among *L*. *habessinicus*, *L*. *fagani*, and *L*. *starcki* from Ethiopia, based on molecular data obtained from phenotypically assigned specimens and their phylogenetic relationships with other *Lepus* species from Africa, Eurasia, and North America. Specifically, we used mitochondrial ATP synthase subunit 6 (ATP6) sequences that have proved useful for molecular phylogenetic resolution at the intraspecific and species levels (Ben Slimen et al., unpubl. data; see also Smith et al.[53]) and intron sequences of one nuclear gene, transferrin (TF) [8]. Given the occurrence of reticulate evolution in several *Lepus* species (see above) and the close phylogenetic relationship between the two mtDNA sequences of *L*. *habessinicus* and *L*. *starcki* published by Pierpaoli et al. [33], we expected introgressive hybridzation or shared ancestral polymorphism in hares from Ethiopia. Thus, we produced microsatellite genotypes for the three species from Ethiopia to obtain indications of genetic species distinction compared to phenotype variation and to evaluate nuclear introgression and extent of contemporaneous gene flow across species (e.g., Andersson et al.[54], Kryger[55], Thulin et al.[56], Suchentrunk et al.[40,41], Liu et al.[9], Wu et al.[27], Melo-Ferreira et al.[10]).

Specifically, we expected distinct phylogenetic divergence between the three species with potential marginal introgressive hybridization: a) *L*. *habessinicus* is conventionally considered closely related to *L*. *capensis* sensu lato or even conspecific with it [46, 1,2,57,58]; b) *L*. *fagani* is considered closely related to the *L*. *saxatilis*/*victoriae* group (e.g., Petter[46], Azzaroli-Puzzetti[57,58], Angermann[2], Flux and Angermann[5]; Petter[59], however, considered it as a subspecies of *L*. *habessinicus*; c) *L*. *starcki* may be a relict form closely related to *L*. *europaeus* [46, 59, 1,2, 5] or only a subspecies of *L*. *capensis* [57,58].

## Materials and methods

### Specimens, phenotypic and taxonomical assignments

**Fig 1:**
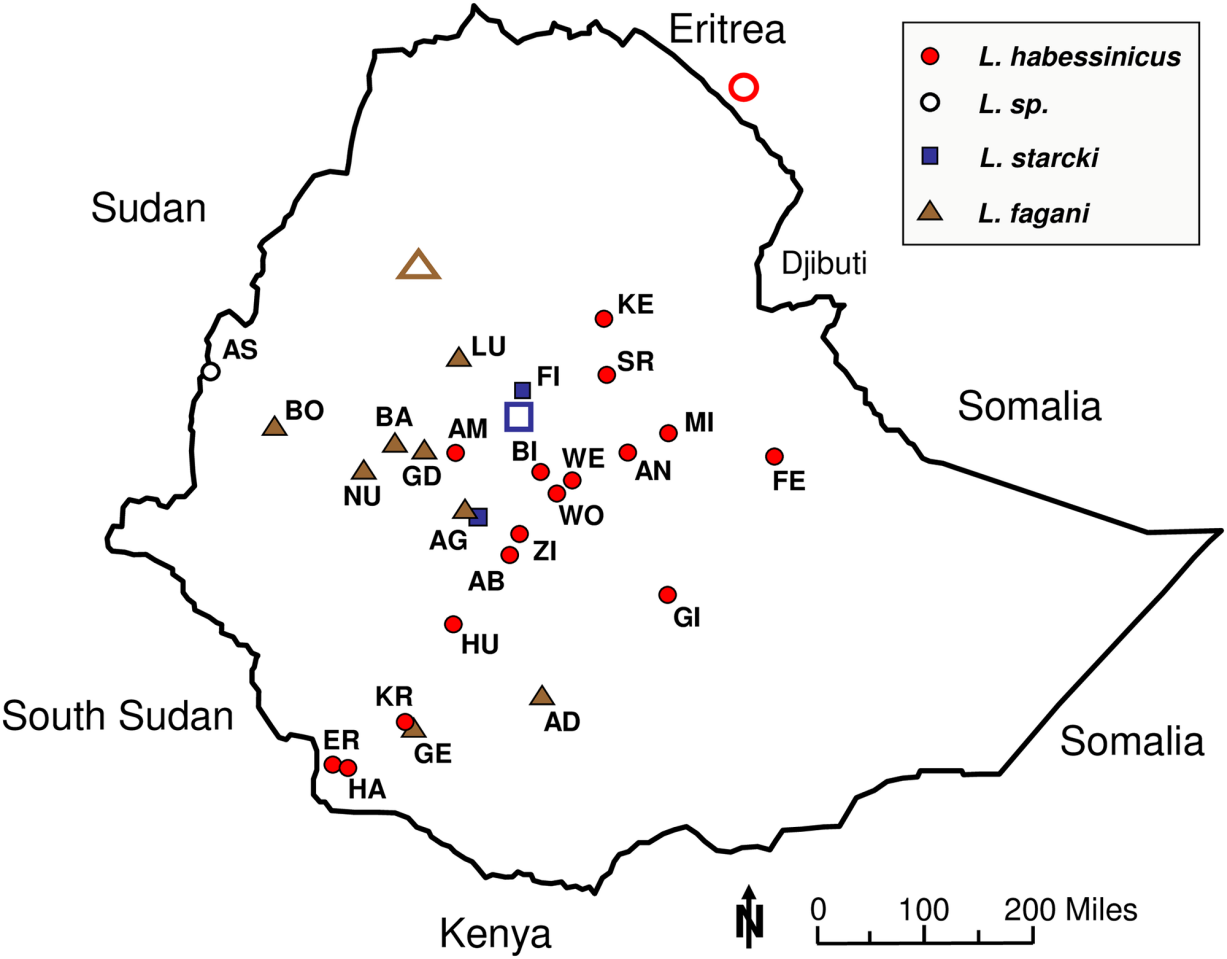
Geographical sample distribution. Full red circles–*Lepus habessinicus*, full brown triangles–*L*. *fagani*, full blue squares–*L*. *starcki*. Open symbols indicate geographical positions of respective holotypes; also given are acronyms of sample localities (for details see Table 1).

We based our study on 120 hares collected from 26 locations in Ethiopia (Fig 1) between February 2010 and December 2012 and on 47 hares of three other *Lepus* species from North and South Africa, the Anatolian Peninsula, and Europe (cape hare, *L*. *capensis* sensu lato, scrub hare, *L*. *saxatilis*, brown hare, *L*. *europaeus*) with supposed close phylogenetic relationships to hares from Ethiopia. All hare samples used in this study were collected with the permit and approval (No. 163/2008) given by the Ethiopian Wildlife Conservation Authority (EWCA) to Zelalem Tolesa (ZT; 2009 to 2014) in the context of his PhD work. The hares were shot by professional hunters based on the permission stated above and tissue samples were collected for further molecular analyses. For outgroup comparison we used two Swiss mountain hares (*L*. *timidus varronis*) that were considered evolutionarily divergent from all the species mentioned above (e.g., Pierpaoli et al.[33], Alves et al.[24]). For some few individuals, however, not all molecular data could be obtained, due to insufficient sample quality (see Table 1 for details). For phylogenetic sequence comparison of our presently obtained TF sequences, we included all reliable TF sequences of 18 *Lepus* species from GenBank.

**Table 1:** Hare samples used in this study.

Taxa	Common name	Collection locality	TF sequences (n = 159)	ATPase 6 sequences (n = 153)	Microsatellites (n = 107)
*Lepus habessinicus*	Abyssinian Hare	Ethiopia, Bishoftu/ Debrezeit (BI)	2	3	2
		Ethiopia, Wonji(WO)	3	3	3
		Ethiopia, Wellenchiti(WE)	5	6	6
		Ethiopia, Andido/Afar (Aw)	4	4	4
		Ethiopia, Miesso (MI)	6	7	6
		Ethiopia, Fedis(FE)	11	11	11
		Ethiopia, Ginnir (GI)	1	1	0
		Ethiopia, Shoa Robit (SR)	2	2	2
		Ethiopia, Kemise (KE)	1	1	1
		Ethiopia, Ziway (ZI)	16	15	16
		Ethiopia, Abijata Shalla NP(AB)	3	3	3
		Ethiopia, Humbo/Wolaita (HU)	4	4	4
		Ethiopia, Gewada/Konso(GE)	0	1	0
		Ethiopia, Erbore/ South Omo (ER)	1	1	1
		Ethiopia, Hamer/South Omo (HM)	1	1	0
*L*. *starcki*	Ethiopian Highland Hare	Ethiopia, Fiche/ Oromia (FI)	26	26	25
		Ethiopia, Agena /Gurage (AG)	1	1	1
*L*. *fagani*	Ethiopian Hare	Ethiopia, Ambo(AM)	0	1	1
		Ethiopia, Gedo(GE)	3	1	2
		Ethiopia, Bako(BA)	1	1	1
		Ethiopia, Nunnu Kumba (NU)	1	1	1
		Ethiopia, Boji Chokorsa (BO)	1	1	1
		Ethiopia, Adolla/ Guji (AD)	8	5	8
		Ethiopia, Lumama/ Gojam (LU)	2	2	2
		Ethiopia, Kerkerty / Konso (KR)	6	0	5
		Ethiopia, Agena /Gurage (AG)	1	1	1
*Lepus sp*.		Ethiopia, Assosa (AS)	0	1	0
*L*. *capensis*	Cape Hare	RSA/South Africa	6	15	0
		Egypt	5	5	0
		Tunisia	6	8	0
*L*. *europaeus*	Brown Hare	Austria	2	2	0
		Anatolian Peninsula	2	2	0
		Bulgaria	2	2	0
		Germany	4	8	0
*L*. *saxatilis*	Scrub Hare	RSA/Western Cape, South Africa	15	5	0
*L*. *timidus*	Mountain Hare	Switzerland	2	2	0
*Oyrctolagus cuniculus*	European rabbit	Austria*	5	0	0

*Wild animals from Germany kept in the breeding station of the Research Institute of Wildlife Ecology, University of Veterinary Medicine Vienna, Austria

We identified species collected in Ethiopia from their composite phenotypes, i.e., coat colour and coat pattern, diagnostic features of ears, skulls, dental characters (see Petter[59], Angermann[1], Azzaroli-Puccetti[57,58], Yalden et al.[60,61]). In addition, we compared our specimens with museum material at “La Specola” (Museo Naturale, Florence, Italy) and the Museum für Naturkunde (Berlin, Germany); the latter holds the holotype of *L*. *habessinicus*. We followed the taxonomy of Flux and Angermann [5], Hoffmann and Smith [12], and Alves and Hackländer[13], who provisionally consider at least three hare species (*L*. *fagani*, *L*. *habessinicus*, *L*. *starcki*) presently occurring in Ethiopia. *Lepus cap*ensis and *L*. *victoriae* may also be present in Ethiopia, but as to our information no confirmed records are available. Similarly, we based species identification of all other individuals of which we currently produced sequences (Table 1) on their external phenotypes, skull and dental characters, range information (e.g., Petter[46,59], Robinson[62], Robinson and Dippenaar[51], Angermann[1], Flux and Angermann[5], Hoffmann and Smith[12], Alves and Hackländer[13], Collins[50], earlier molecular data (Sert et al.[63,64], Ben Slimen et al.[38, 52, 21, 31,37], Kryger[55], Kasapidis et al.[14], Stamatis et al.[65, 15], Suchentrunk et al.[6], Suchentrunk et al.[40,41]), as well as on the provisional *Lepus* taxonomy [5, 12, 13, 49].

### Laboratory procedures

#### DNA extraction, PCR amplification, and sequencing

We used the GenElute^™^ Mammalian Genomic DNA Miniprep kit (Sigma-Aldrich) to extract total genomic DNA from ear tissues and followed Smith et al. [53] to PCR amplify a 416 bp segment of the mitochondrial ATPase sub-unit 6 (ATP6, from site 8142 to 8594; see also Arnason et al.[66]). We amplified a 434 bp fragment of the transferrin gene (TF, between exons 6 and 7) according to Alves et al. [8] and included TF sequences of five rabbits (*Oryctolagus cuniculus*) from the breeding station at the Wildlife Research Institute of the University of Veterinary Medicine in Vienna (Austria). Moreover, we retrieved 111 TF sequences of 13 *Lepus* species plus one European rabbit two of *Sylvilagus floridanus* from GenBank for phylogenetic comparison (S1 Table). However, our ATP6 primers did not yield satisfactory results for the *Oryctolagus* samples, and no ATP6 sequences were available for *Sylvilagus* on GenBank.

We used the enzymatic clean-up process using Exonuclease I and Shrimp Alkaline Phosphatase (Werle *et al*.[67]) for PCR product purification and performed cycle sequencing of both strands with an ABI 3130xl genetic analyzer. We used BioEdit vers. 7.1.3.0 (Hall[68]) for sequence alignment and checked alignments by eye. We used composite sequences as obtained from both strands for phylogenetic analyses. We used the Phase 2.1.1 algorithm [69,70], as implemented in DnaSP vers. 5.10.01 Labrado and Rozas[71], to reconstruct haplotypes (e.g., Flot et al.[72], Garrick et al. [73]) for TF sequences with more than one ambiguity.

### Phylogenetic analyses of ATP6 and TF sequences

We used DnaSP to calculate numbers of variable sites, phylogenetic informative sites and singletons as well as haplotype (h) and nucleotide (π) diversity, and mean numbers of pairwise differences (k) to indicate levels of polymorphism.

We constructed phylogenetic haplotype networks for both sets of sequences by using the median-joining (MJ) algorithm (with default parameter settings) implemented in the Network software, version 4.6.1.1 [74]. Contrary to tree-based inference, networks can uncover possible alternative evolutionary pathways of haplotypes; hence, the depiction of evolutionary relationships among haplotypes is not reduced to bifurcating events and displaying taxa only at terminal positions. This may facilitate inference on the presence of shared ancestral polymorphism or recent introgression, particularly for clusters with low bootstrap support (e.g., Hall [75], Morrison[76]).

In addition, we used MEGA vers. 5.0 Kumar et al.[77], Tamura *et al*.[78] to analyze phylogenetic relationships among haplotypes by Neighbor Joining (NJ), Saitou and Nei[79], Tamura and Kumar[80] and Maximum Likelihood (ML), Kimura[81] modelling. We also included 111 TF seqeuences of 18 *Lepus* species from different parts of the world, one European rabbit and two *Sylvilagus floridanus* (Eastern Cottontail Rabbit) sequences, downloaded from GenBank, but accepted only those with a maximum of one nucleotide base ambiguity in our alignment, in order to exclude the chance of picking up problematic ones. Prior to NJ analyses, we checked sequences for suitability by calculating average Jukes Cantor (JC) distances (criterion: average pairwise JC distance < 1.0; Nei and Kumar[82], see e.g., Hall[75]). We used the Maximum Composite Likelihood model for NJ trees, as recommended by Kumar et al. [77], (also Hall[75]), and assessed confidence in resulting nodes by 1000 bootstrap replicates. For ML analyses, we applied the most appropriate evolutionary model based on BIC values (e.g., Posada[83]), according to the model test option in MEGA with 1000 bootstrap replicates. We also used MEGA to calculate net mean between group p distances for ATP6 and TF sequences.

We used MrBayes v. 3.2 Ronquist et al.[84], that estimates phylogenetic relationships by seeking the most likely tree given our actual sequence data and the chosen substitution model. We began our Bayesian inference (BI) with random starting trees and ran it for 10 million generations, with Markov chains sampled every 1000 generations. We checked the average standard deviation of split frequencies for parameter convergence. The first 2.5 million generations were excluded as burn-in. We conducted the BI twice to ensure that the analyses were not trapped in local optimum [85,86]. The remaining trees from both analyses were used to create a majority rule consensus tree where the percent of samples recovering the same clade represented the posterior probability value of that clade. For tree viewing and editing we used FigTree v.1.4.2 [87].

### Microsatellite screening

To investigate the contemporary overall genepool partitioning among the three species in Ethiopia and possible ongoing migration among them, we screened 59 *L*. *habessinicus*, 26 *L*. *starcki*, and 22 *L*. *fagani* for allelic variation at the following thirteen microsatellite loci: Sol08, Sol28, Sol30 Rico et al.[88], Sol33 Surridge et al.[89], Sat 2, Sat 8, and Sat 12 Mougel et al.[90], Lsa 1, Lsa 2, Lsa 3, Lsa 6 and Lsa 8 Kryger[55], and INRACCDDV0001 (“Inra1”) Chantry-Darmon et al.[91]; for PCR details see also Ben Slimen et al.[37] and Suchentrunk et al. [40,41]. PCR products were electrophoresed on a LI-COR 4200 automated sequencer along with fluorescently labelled size standard (30–350 bp sizing standard; LI-COR^®^ Biotechnology Division) and allele lengths were determined by the Gene ImageIR ver. 3.52 software (LI-COR, Inc., 1990–1998). We used MICRO-CHECKER 2.2. [92] for maximum likelihood estimates of the presence of null alleles and associated frequencies.

#### Population genetic statistics

Allelic variability, Hardy Weinberg, and Linkage disequilibrium

We calculated allele frequencies, mean number of alleles (A), observed (Ho) and expected (He) heterozygosity for each locus and species with GENETIX 4.05.2 Belkhir [93]. We tested each locus, separately for each species, for deviation from Hardy-Weinberg equilibrium (HWE) and pairs of loci for linkage disequilibrium (LD) using the Markov chain method implemented in GENEPOP version 3.4 (Raymond and Rousset[94], with default parameter settings and accounted for multiple tests by strict Bonferroni corrections at α = 0.05 Rice[95],; the latter was used for all further test series. Due to a significant LD signal in *L*. *habessinicus* (see Results), we excluded the Lsa2 locus from all further calculations. We calculated species-specific Weir and Cockerham[96] estimators of population level “inbreeding coefficients” due to non-random mating (*F*_*is*_) and associated significance levels for deviation from zero by permutation tests (10.000 permutations) by using GENETIX.

Evaluating possible homoplasy

We could not exclude size homoplasy, particularly at highly polymorphic loci (e.g., Garza and Freimer[97]; but see Ben Slimen et al.[37], for hares from Europe and Africa). Under pronounced homoplasy, genetic differentiation is expected to be higher in lesser polymorphic loci (e.g., Ben Slimen et al.[37]). We used S-PLUS 6.2 [98] to run a generalized least squares regression model (gls) to test locus-specific *f*_*st*_ values (across the three Ethiopian species, respectively) for variation due to locus-specific numbers of alleles and sample sizes (fixed factors), by accounting for occasionally missing data. Significantly higher *f*_*st*_ values due to lower numbers of alleles per locus would concord with a homoplasy effect. Additionally, we counted genotypes shared by two or all three species, separately for each locus. Low genotype sharing among species would suggest negligible homoplasy, particularly, if the shared genotypes occurred either at low or at very divergent frequencies among species.

Testing for reduced effective population size

We used BOTTLENECK vers. 1.2.02 Cornuet and Luikart[99], Luikart and Cornuet[100],) to run one-tailed Wilcoxon sign rank tests (10.000 iterations), separately for each species, to test for reduced effective population size in the recent past that could have influenced genetic variability and differentiation among the Ethiopian species. Thereby, we applied the stepwise mutation model (S.M.M), the infinite allele model (I.A.M.), and a two-phased model (T.P.M., with default settings) of microsatellite evolution. Additionally, we calculated the mean ratio of the number of alleles to the range of allele size (M ratios) separately for each species, to infer possible genetic bottlenecks in the more distant past[101].

Genetic differentiation and contemporary introgression

We calculated pariwise *F*_*st*_ values of relative genetic differentiation between species and tested significance by 10.000 permutations by using GENETIX. We used GeneAlex v. 6.5 [102] to calculate pairwise *G´*_*st*_ Hedrick[103], and *D*_est_ (i.e., Jost´s D; Jost[104]) and ssociated significance levels (10.000 randomizations); the latter two indices account for high heterozygosity, whereas *F*_*st*_ does not, which may underestimate differentiation. Moreover, we used MSA 4.05 [105] to calculate pairwise Cavalli-Sforza and Edwards chord (CSE) distances and constructed a NJ dendrogram using PHYLIP 3.6.9.5[106]. Individuals clustering with non-conspecifics might highlight recently introgressed individuals. Further, we used Arlequin v. 3.11 [107] for an analysis of molecular variance (AMOVA) among the three species.

We used GENECLASS 2 [108] to determine the likelihood of an individual being a “first generation migrant” between the phenotypically determined species using the criterion of Paetkau et al.[109] and the Bayesian simulation algorithm of Paetkau et al.[110] with 10.000 simulated individuals and default allelic frequencies of 0.01. “First generation migrants” detected within a phenotypically determined species would in principle indicate incorrect phenotypic species determinations or individuals being massively introgressed by genes of a different species. In addition, we estimated the likelihood of an individual’s multilocus genotype to be assigned to the phenotypically determined species from which it was sampled by the Bayesian method of Rannala and Mountain [111] and the resampling (100.000 simulated indivuduals) algorithm of Paetkau et al.[109].

We used STRUCTURE [112,113] to assess the most likely number of population groupings (i.e., k genetic clusters) compatible with the observed genotypic distribution across all samples. We run models for all 107 individuals based on 12 loci, without and with prior population (i.e., species) information under admixture models and correlated allele frequencies. The likelihood when assuming different numbers of genetic clusters (k = 1 to 11) was calculated by 150.000 MCMC following a burn-in of 50.000, and ten iterations per k. Mean and standard deviation of likelihood values were calculated for each k and plotted together with Evanno´s [114] ad hoc statistic by using the STRUCTURE HARVESTER on-line option [115]. The model runs were repeated with only eight loci (i.e., excluding Sol33, Lsa3, Sat2, Sat8), as null alleles at higher frequencies (up to 30%) could not be excluded for at least one species at those loci.

We used MIGRATE v.3.0[116] for coalescent-theory based ML estimates of current migration rates between the three species, as additional indication of contemporary hybridization between species. We used the following specifications for our “Brownian Motion” model assuming constant mutation rates for 12 loci: 10 short chains with 5.000 recorded steps, 500.000 visited genealogies, burn-in of 10.000; 2 long chains with 50.000 recorded steps and 10.000.000 visited genealogies; for the set of eight loci (see above) the specifications were: 10 short chains with 1.000 steps, 200.000 visited genealogies, burin-in of 10.000; 2 long chains with 1.000 recorded steps and 100.000 visited genealogies.

## Results

### External phenotypes and species determination

All hares collected in Ethiopia, except for one neonate from the Abjiata Shala National Park (AB) and one subadult from Assosa (AS, Fig 1) could be unambiguously assigned phenotypically to one of the following three species: Ethiopian Hare, *L*. *fagani* Thomas, 1902; Abyssinian Hare, *L*. *habessinicus* Hemprich and Ehrenberg, 1832; Ethiopian Highland Hare, *L*. *starcki* Petter, 1963 [1, 60, 5]. The subadult specimen from AS featured external characteristics of *L*. *fagani*, but it´s partly damaged skull and incomplete external phenotype did not permit a definitive phenotypic determination. All our *L*. *habessinicus* specimens matched the form “*L*. *tigrensis*” (synonym to *L*. *habessinicus*; Angermann [1], Yalden et al.[60], Flux and Angermann[5]), albeit with several individuals displaying somewhat enlarged blackish areas on the outer pinnae, instead of narrow rims as e.g. depicted in Yalden et al.[60]. Our *L*. *fagani* and *L*. *starcki* specimens matched the species descriptions of Angermann [1], Yalden et al. [60], and Flux and Angermann [5]. No phenotypes matching the forms “*L*. *crawshayi*” or “*L*. *cordeauxi*” (Yalden et al.[60], were collected presently. However, we noticed similarities between the presently examined phenotypes (external, skull morphometry, dental characters; unpubl. data) and forms of East African cape hares, *L*. *capensis* L., 1758 or savanna hares, *L*. *victoriae* Thomas, 1893 as described by Flux and Flux [117] and the forms described by Azzaroli-Puccetti [57,58].

### Mitochondrial ATP6 sequence variation and phylogenetic relationships

We obtained ATP6 sequences of 104 hares from Ethiopia (*L*. *habessinicus*, *L*. *starcki*, *L*. *fagani*) and of 49 specimens of four species (*L*. *capensis*, *L*. *saxatilis*, *L*. *europaeus*, *L*. *timidus*) from Africa, the Anatolian Peninsula, and Europe (Table 1). All new variable sequences were deposited on GenBank (see also S2 Table). All sequences could be translated into proteins (hence, no suspect of pseudogenes) and the composite alignment encompassing 416 base pairs revealed 64 haplotypes. Overall haplotype diversity (h) was 0.952, overall nucleotide diversity (π) was 0.054, and average number of nucleotide difference (k) was 22.36.

**Fig 2:**
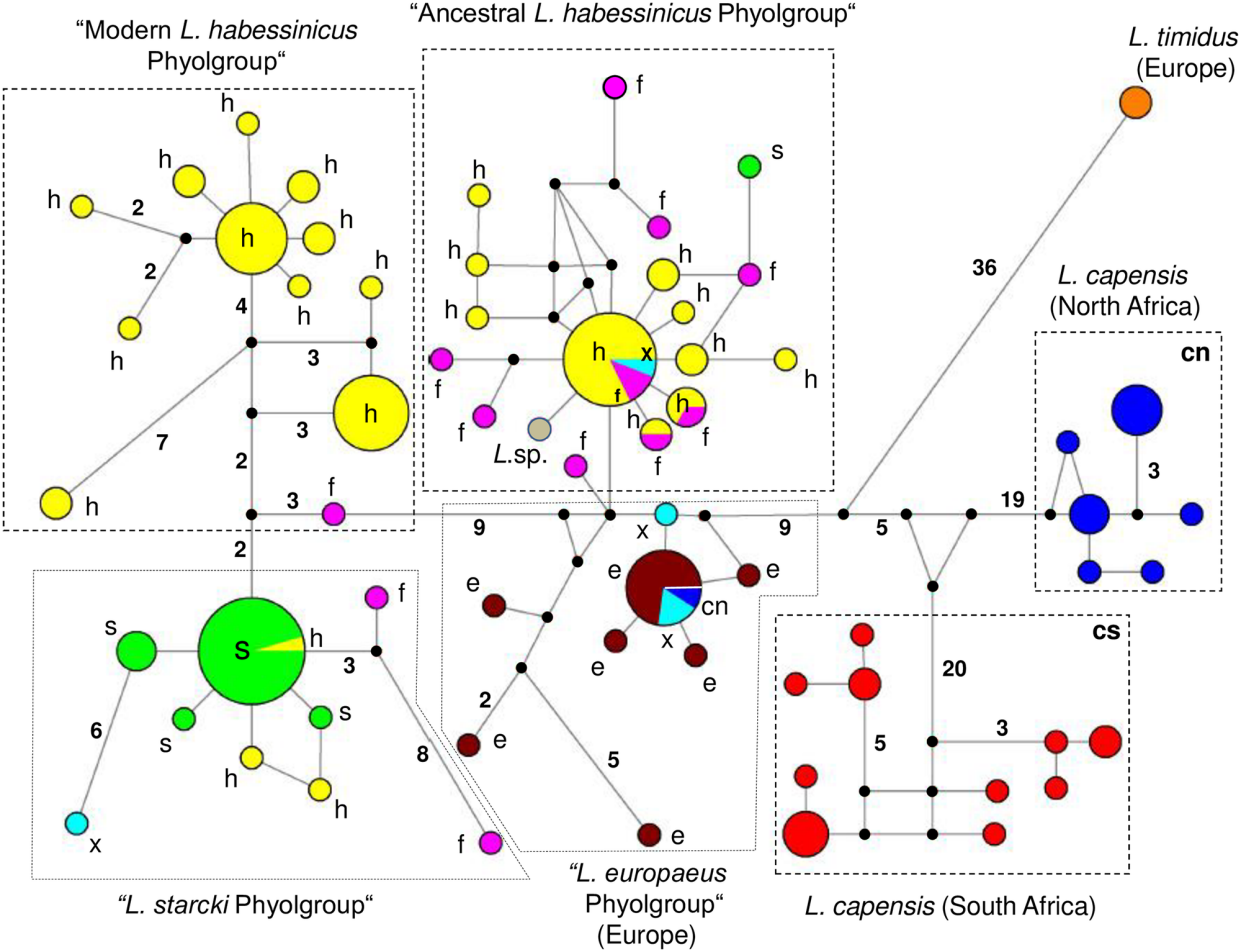
Median joining network of ATP6 haplotypes. Haplotypes (pies) are proportional to total sample number, taxon assignments of single haplotypes (pie slices) represent percentages of taxa per haplotype. Black dots indicate inferred haplotypes, not revealed presently, numbers associated with lines give numbers of substitutions between any two haplotypes/inferred haplotypes, if more than one (single mutational steps between any two haplotypes are not indicated). Evolutionary distances between haplotypes are only roughly in proportional scale. Taxa acronyms: cn–*Lepus capensis*, North Africa, cs–*L*. *capensis*, South Africa, e–*L*. *europaeus*, f–L. *fagani*, h–*L*. *habessinicus*, s–*L*. *starcki*, x–*L*. *saxatilis*, *Lsp*.–phenotypically undetermined hare specimen.

The median joining network (Fig 2) grouped all North and South African cape hares clearly separate from all other hares. The two *L*. *timidus* sequences resulted in one haplotype at a position far distant from all hares studied presently, and connected to a node between the South and North African *L*. *capensis* phylogroups on the one hand and all other hares studied on the other. For *L*. *habessinicus* two phylogroups were revealed, phylogroup A consisting of haplotypes from southwestern Ethiopia and the southern Rift Valley in Ethiopia, and phylogroup B comprising haplotypes from central and eastern Ethiopia, and the northern Rift Valley in Ethiopia. Phylogroup A was basal to phylogroup B, as revealed by their positions relative to *L*. *timidus* and *L*. *capensis* in the network. Phylogroup A, however, also contained sequences of eight *L*. *fagani*, one *L*. *starcki*, one *L*. *saxatilis*, and one morphologically unidentified subadult *Lepus* individual from western Ethiopia (AS, see Fig 1). Except for the one *L*. *starcki* sequence in phylogroup A, all other *L*. *starcki* sequences clustered into a single–evolutionarily young–phylogroup (“*L*. *starcki* phylogroup”) closely related to phylogroup B of *L*. *habessinicus*. Most sequences of *L*. *fagani* clustered into phylogroup A of *L*. *habessinicus*, two clustered with the *L*. *starcki* phylogroup, and one sequence represented a single haplotype between phylogroup A of *L*. *habessinicus* and the *L*. *starcki* phylogroup. Three *L*. *habessinicus* sequences clustered within the *L*. *starcki* phylogroup. All 14 *L*. *europaeus* sequences clustered into two closely related phylogroups, a European and an Anatolian Peninsula phylogroup. Neither the Ethiopian *L*. *fagani* nor the South African *L*. *saxatilis* sequences formed distinct phylogroups; rather, they were scattered across three phylogroups, two Ethiopian (phylogroup A of *L*. *habessinicus*, *L*. *starcki* phylogroup) and the European phylogroup, consisting of *L*. *europaeus* sequences from Europe.

**Fig 3:**
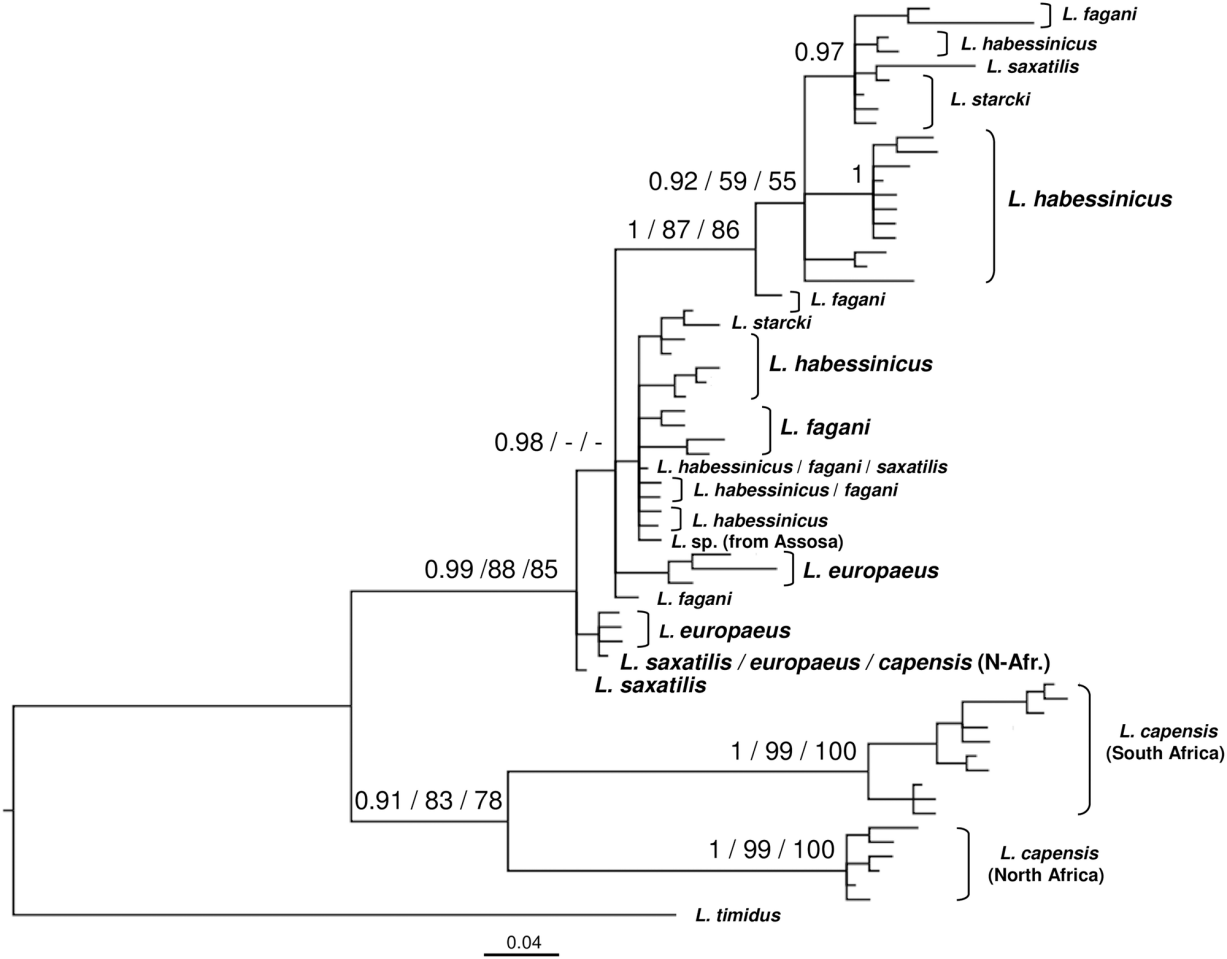
Bayesian dendrogram of mtATP6 haplotypes. Node support above 50% is given for Bayesian Inference, ML, and NJ analyses, respectively. For details see “Material and methods”.

Among all 125 polymorphic sites, 17 were singletons, and 108 were phylogenetically informative. Under the BIC criterion, the most appropriate model of sequence evolution was the HKY+G (Γ = 0.31). A matrix of net mean between group (i.e., species) p distances for all 153 hares sequenced presently is displayed in S3 Table. The BI, ML, and NJ analyses produced in essence concordant tree topologies. The average standard deviation of the split frequencies of the BI was 0.004 when runs were added. The BI majority-rule consensus tree is shown in Fig 3 along with the posterior probabilities for nodes and the bootstrap values for the ML and NJ analyses superimposed.

### Transferrin (TF) sequence variation and phylogenetic relationships

We obtained TF sequences of 154 hares (110 hares from Ethiopia, 44 hares of four *Lepus* species from Africa, Asia Minor, and Europe and of five European rabbits (*Oryctolagus cuniculus*) (Table 1). All new variable TF sequences are available from GenBank (for accession numbers and details see S4 Table and for pairwise net p distances among taxa see S5 Table). Among our TF sequences, we observed one insertion (1bp) in four *L*. *starcki*, in ten *L*. *europaeus*, and in the two *L*. *timidus* at the same position as in several of the downloaded sequences. Our presently produced sequences had a maximum of five ambiguities, and resulted in an overall alignment length of 427 base pairs, when the downloaded sequences were included. The overall alignment included six indels with a total length of 17 sites. However, all were excluded from phylogenetic analyses. Phasing of the overall alignment resulted in 92 haplotypes without ambiguous resolution and 110 polymorphic sites. Overall h was 0.942, π amounted to 0.02, and k was 8.15.

**Fig 4:**
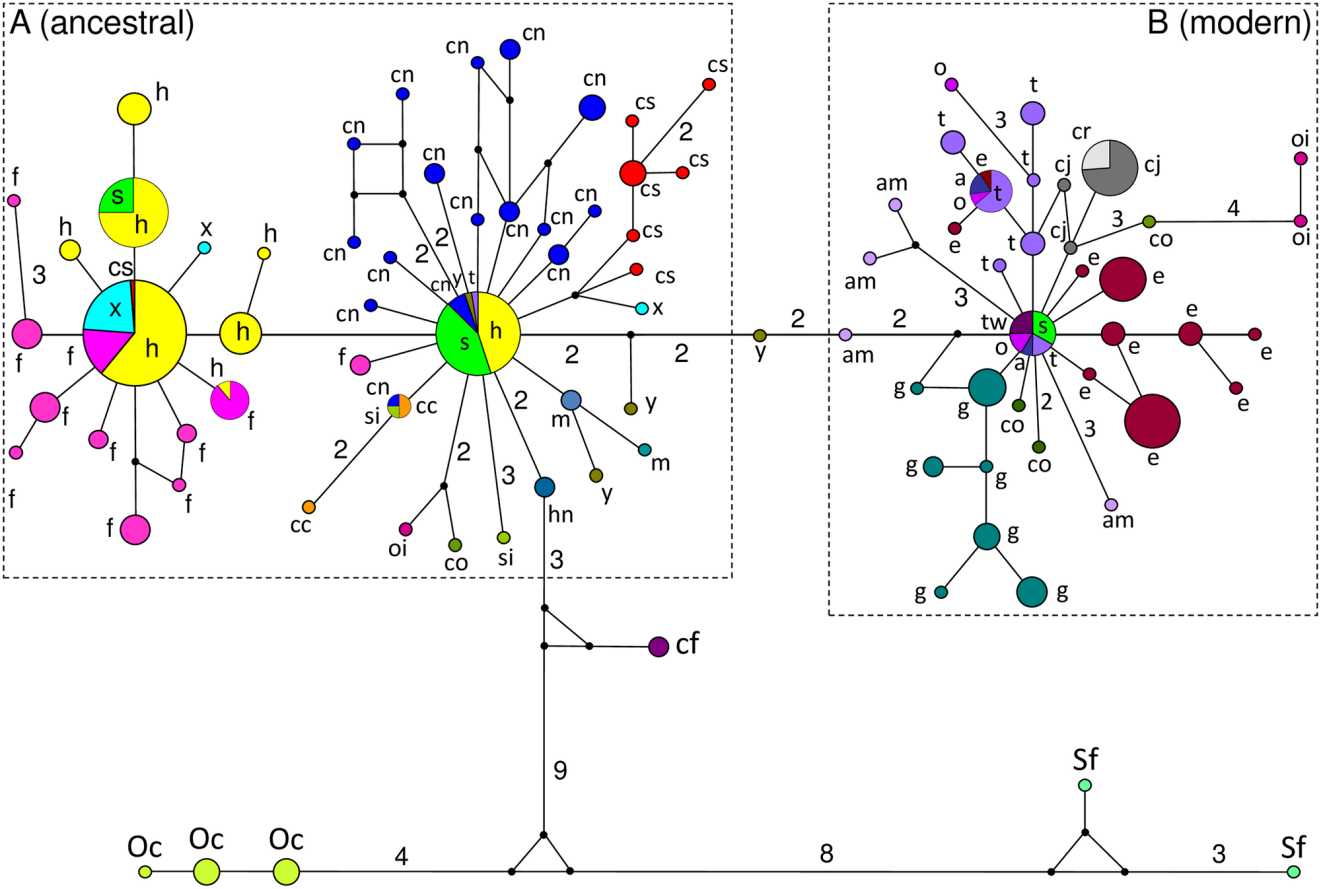
Median joining network of TF haplotypes. Haplotypes (pies) are proportional to the total sample number, taxon assignments of single haplotypes (pie slices) represent percentages of taxa per haplotype. Black dots indicate inferred haplotypes, not revealed presently, numbers associated with lines give numbers of substitutions between any two haplotypes/inferred haplotypes, if more than one; single mutational steps between any two haplotypes are not indicated. Evolutionary distances between haplotypes are only roughly in proportional scale. Taxa acronyms: cn–*Lepus capensis*, North Africa, cs–*L*. *capensis*, South Africa, f–*L*. *fagani*, h–*L*. *habessinicus*, s–*L*. *starcki*, x–*L*. *saxatilis*, *Lsp*.–phenotypically undetermined hare specimen, cc–*L*. *capensis*, China, co–*L*. *comus*, hn–*L*. *hainanus*, m–*L*. *mandshuricus*, si–*L*. *sinensis*, oi–*L*. *oiostolus*, y–*L*. *yarkandensis*, a–*L*. *arcticus*, am–*L*. *americanus*, cf–*L*. *californicus*, cj–*L*. *castroviejoi*, cr–*L*. *corsicanus*, e–*L*. *europaeus*, g–*L*. *granatensis*, o–*L*. *othus*, t–*L*. *timidus*, tw–*L*. *twonsendii*, sf–*Sylvilagus floridanus*, Oc–*Oryctolagus cuniculus*.

**Fig 5:**
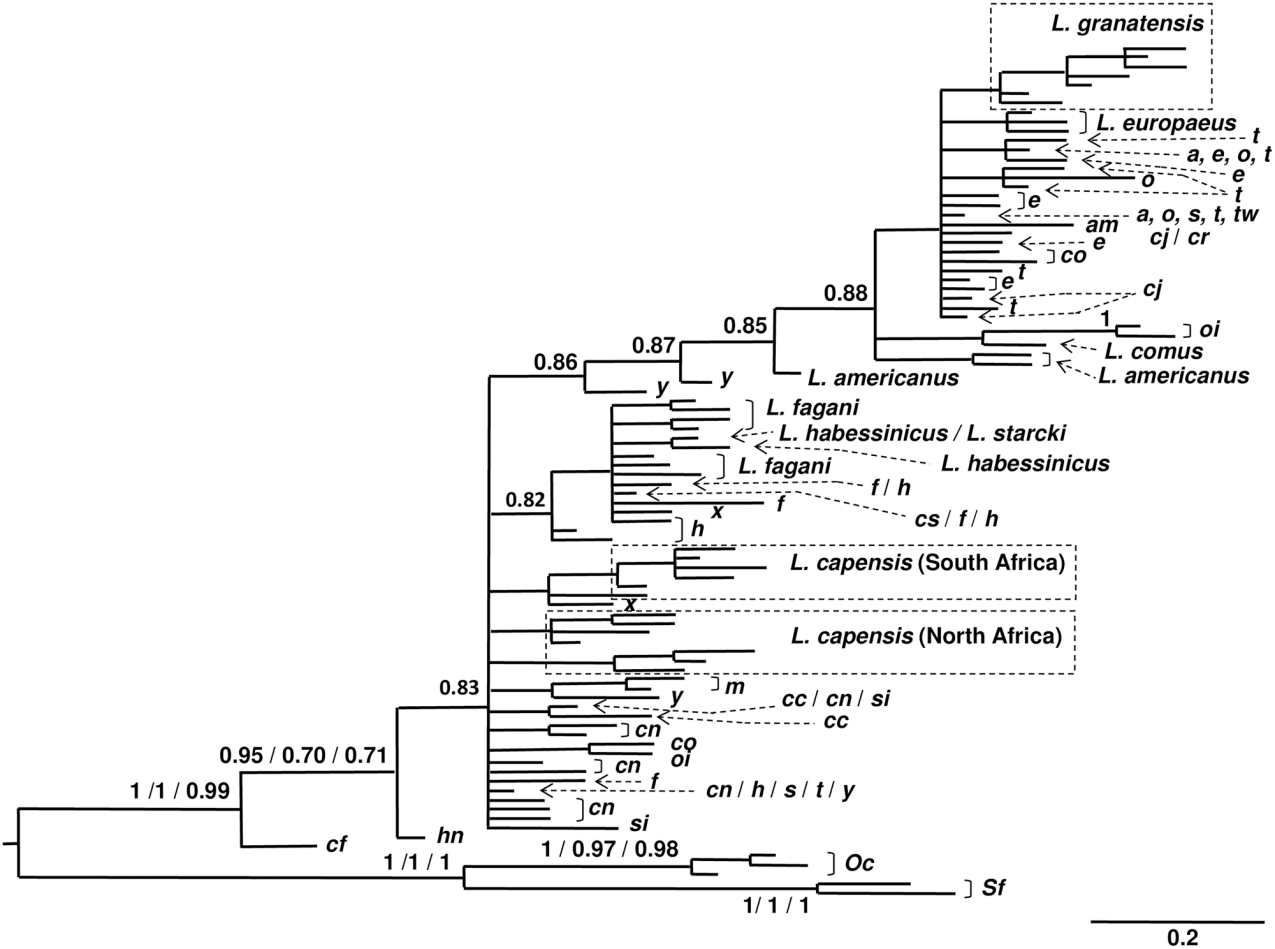
Bayesian dendrogram of TF haplotypes. Node support above 50% is given for Bayesian Inference, ML, and NJ analyses (for details see “Materials and methods” section). Acronyms of taxa: a–*Lepus arcticus*, am–*L*. *americanus*, c–*L*. *comus*, cc–*L*. *capensis*, China, cf–*L*. *californicus*, cj–*L*. *castroviejoi*, cn–*L*. *capensis*, North Africa, cr–*L*. *corsicanus*, cs–*L*. *capensis*, South Africa, e–*L*. *europaeus*, f–*L*. *fagani*, h–*L*. *habessinicus*, hn–*L*. *hainanus*, m–*L*. *mandshuricus*, o–*L*. *othus*, oi–*L*. *oiostolus*, s–*L*. *starcki*, si–*L*. *sinensis*, t–*L*. *timidus*, tw–*L*. *townsendii*, x–*L*. *saxatilis*, y–*L*. *yarkandensis*, Oc–*Orycotlagus cuniculus*, Sf–*Sylvilagus floridanus*.

The network topology reflected only roughly the major geographic origin of the species, with some haplotypes, however, being shared by species from different continents (Fig 4). It was partitioned into two major phylogroups (A and B) in addition to the *Oryctolagus/Sylvilagus* outgroup. Phylogroup A appeared closer to the outgroup than phylogroup B. This was also indicated by the BI tree, whereas the NJ and ML trees did not recover that topology (Fig 5). Phylogroup A contained species of African origin (*L*. *habessinicus*, *L*. *fagani*, *L*. *starcki*, *L*. *capensis* from South and North Africa, *L*. *saxatilis*), as well as species from Eurasia (Chinese *L*. *capensis* sensu lato, *L*. *comus*, *L*. *hainanus*, *L*. *mandshuricus*, *L*. *oiostolus*, *L*. *sinensis*, *L*. *timidus*, *L*. *yarkandensis*) and North America (*L*. *californicus*). Phylogroup B consisted of Eurasian (*L*. *castroviejoi*, *L*. *corsicanus*, *L*. *comus*, *L*. *granatensis*, *L*. *europaeus*, *L*. *timidus*, *L*. *oiostolus*) and North American (*L*. *americanus*, *L*. *arcticus*, *L*. *othus*, *L*. *townsendii*) species, except for a single haplotype shared by four *L*. *starcki* individuals and European and North American species (*L*. *acrticus*, *L*. *othus*, *L*. *timidus*, *L*. *townsendii*). The downloaded *L*. *californicus* and *L*. *hainanus* sequences connected the outgroup haplotypes with a major haplotype of phylogroup A. That latter haplotype was found in *L*. *habessinicus*, *L*. *starcki*, and North African *L*. *capensis*, as well as in *L*. *timidus* and *L*. *yarkandensis*. It gave rise to a number of haplotypes of species of Asian (Chinese *L*. *capensis*, *L*. *mandshuricus*, *L*. *sinensis*, *L*. *yarkandensis*) and African origin (North and South African *L*. *capensis*, *L*. *saxatilis*), and further to a closely related haplogroup, typical for Ethiopian species and including also *L*. *saxatilis* and one *L*. *capensis* from South Africa.

Sequence evolution was best described by the K2+G (Γ = 0.5389) model under the BIC criterion. The overall TF alignment (n = 377 sequences of 159 individuals presently analyzed and 111 individuals from GenBank) revealed 110 variable sites, 77 phylogenetically informative sites, and 33 singletons (for net mean between group p distances see S5 Table). Whereas the BI tree topology (average s.d. of the split frequencies of the BI was 0.007 when runs were added) reflected by and large the network topology; the NJ and ML analyses, however, resulted in somewhat different tree topologies.

### Microsatellite variability, genetic differentiation, recent hybridization

#### Genetic variability, Linkage disequilibrium, Hardy Weinberg equilibrium

Due to a significant (p<0.00001) LD for the Lsa2 locus with the Sol8 and Sat12 loci within *L*. *habessinicus*, the Lsa2 locus was omitted from all further analyses. For the remaining 12 loci a total of 146 alleles was found (overall A = 12.17), ranging from four (Lsa 6) to 22 alleles (Sol 28) per locus. Among all alleles, 68 (46.6%) were private (36.8% for *L*. *habessinicus*, 16.2% for *L*. *starcki*, 47.1% for *L*. *fagani*), 56 (38.4%) were shared between two species, and only 22 (15.1%) were shared among all three species. Frequencies of private alleles ranged between 0.85 and 47.5% (mean = 8.88%, s.d. = 12.6) in *L*. *habessinicus*, 33.85–66.0% (mean = 15.65%, s.d. = 20.1%) in *L*. *starcki*, and between 2.27 and 29.55% (mean = 8.91%, s.d. = 8.3%) in *L*. *fagani*. Locus-specific numbers of alleles (N_a_), allele ranges (R), h_e_, and h_o_ are given separately for each species in Table 2, as well as species-specific H_e_, H_o_, A, numbers of private alleles and F_is_. Significant deviations from HWE were found in each species. Our MICRO-CHECKER runs did not exclude presence of null alleles at the Sol33, Lsa3, Sat8, Sat2 loci for one or more species, with estimated frequencies exceeding somewhat 20%. Null alleles with frequencies up to ca. 20–30% do not seriously confound population genetic results Chapuis and Estoup[118]; however, for confirmation of the results based on 12 loci, we repeated several analyses (Bottleneck, Geneclass, Migrate, M ratios, Structure) by excluding the Sol33, Lsa3, Sat2, Sat8 loci.

**Table 2:** Microsatellite variability of the three hare species in Ethiopia.

							Locus Name							
Species		Sol08	Sol28	Sol30	Sol33	Inra1	Sat2	Sat8	Sat12	Lsa1	Lsa3	Lsa6	Lsa8	F_IS_
*L*. *habessinicus* (26)	Na	9	14	14	5	5	11	12	7	7	7	3	7	0.229*
A = 8.42	R	124–140	151–189	151–197	213–221	200–208	223–249	95–119	102–134	164–176	192–218	166–170	182–194	
H_e_ = 0.636	he	0.798	0.827	0.888	0.538	0.084	0.765	0.779	0.681	0.702	0.804	0.097	0.667	
H_o_ = 0.496	ho	0.69	0.655	0.776	0.339	0.086	0.569	0.509	0.603	0.593	0.414	0.102	0.61	
*L*. *starcki* (11)	Na	3	3	4	5	6	7	7	4	2	6	3	5	0.154*
A = 4.583	R	116–132	143–169	171–195	213–225	208–218	225–243	95–121	102–126	164–174	198–216	164–168	186–194	
H_e_ = 0.494	he	0.544	0.49	0.241	0.49	0.722	0.45	0.592	0.481	0.286	0.702	0.269	0.657	
H_o_ = 0.427	ho	0.654	0.36	0.154	0.462	0.654	0.346	0.48	0.385	0.269	0.44	0.154	0.769	
*L*. *fagani* (31)	Na	14	14	13	6	3	8	5	8	4	7	2	8	0.229*
A = 7.667	R	102–136	157–193	155–187	207–221	202–206	221–253	93–111	102–134	160–166	184–208	164–168	178–196	
H_e_ = 0.685	he	0.885	0.883	0.892	0.779	0.373	0.861	0.524	0.793	0.679	0.794	0.095	0.663	
H_o_ = 0.545	ho	1	0.727	0.682	0.727	0.273	0.462	0.286	0.773	0.773	0.286	0.1	0.5	

Na–number of alleles per locus, R–allelic size range in bp, he–locus-specific expected heterozygosity, ho–locus-specific observed heterozygosity, separately for each species, He—species-specific expected heterozygosity, Ho–observed species-specific heterozygosity, A–mean number of alleles per locus, F_IS_−species-specific “inbreeding coefficient”. The number of private alleles for each species is given in parentheses associated with the species name. Significant deviation from HWE is indicated by * with F_IS_.

#### Homoplasy signal

Our gls model indicated only a tendency for slightly higher f_st_ values (across the three species) for lesser polymorphic loci compared to higher polymorphic loci, when accounting for varying sample sizes (coefficient = -0.0233, F_1,7_ = 3.813, p = 0.083). That tendency was, however, driven by only one locus (Lsa 6). Moreover, our genotype comparisons between the species revealed that only 18.7% of all 337 recovered genotypes at 12 loci were shared by two species, and only 1.8% was shared by all three species. Mean species-specific frequencies of shared genotypes at highly polymorphic loci (37–51 genotypes) averaged at 8.3% (median = 6.6%, range: 2.9–42.1%). For lesser polymorphic loci (6–30 genotypes) the frequencies averaged at 12.5% (median = 7.8%, range: 2.4–77.5%). At Sol30, the locus with most (51) genotypes, only 9.8% of all genotypes were shared by two species, with a mean species-specific frequency of 13.0%, a median of 6.6%, and a range between 4.8 and 42.1%; the maximal value of 42.1% resulted from the very high frequency (80.8%) of a genotype in *L*. *starcki* but a very low corresponding frequency (3.4%) in *L*. *habessinicus* (suggesting also no homoplasy problem). Overall, potential homoplasy seemed to be of no major concern for our data as already observed by Ben Slimen et al. [37].

#### Reduction of genetic population size

Wilcoxon sign rank tests did not return any significant signals of a recent bottleneck, when accounting for multiple tests. The M ratios (*L*. *habessinicus*: 0.786/0.788; *L*. *starcki*: 0.56/0.518; *L*. *fagani*: 0.7/0.738 for 12 and 8 loci, respectively), however, indicated a reduction of the effective population size for *L*. *starcki* in the more distant past.

Genetic differentiation, molecular species assignment, recent introgression

Overall F_ST_ was 0.275 (95% c.i.: 0.208–0.364); Table 3 displays pairwise F_st_ values between species, along with pairwise G´_st_ and Jost´s D. The AMOVA revealed significant partitioning of relative genetic variability among the three species at 27.44% (variance comp. = 1.360, p < 0.00001), among individuals within species at 14.3% (variance comp. = 0.709, p < 0.00001), and within individuals at 58.26% (variance comp. = 2.890, p < 0.00001). Within *L*. *habessinicus*, only 3.59% of relative genetic variability were due to partitioning between the ancestral and the recent ATP6 phylogroups A and B.

**Table 3:** Genetic differentiation and migration between *Lepus* species from Ethiopia.

A		12 loci		B			
		*L*. *sta*	*L*. *fag*		*L*. *hab*	*L*. *sta*	*L*. *fag*
*L*. *hab*		0.350*	0.142*	*L*. *hab*		1.457 (1.408–1.506)	0.311 (0.302–0.32)
		0.814*	0.384*			1.319 (1.257–1.383))	0.43 (0.417–0.444)
		0.761*	0.334*				
*L*. *sta*	0.200**		0.332*	*L*.*sta*	1.595 (1.539–1.651)		0.323 (0.314–0.332)
			0.795*		1.128 (1.069–1.188)		0.268 (0.258–0.28)
			0.745*				
*L*. *fag*	0.138**	0.232**		*L*.*fag*	2.447 (2.38–2.516)	2.019 (1.962–2.078)	
					3.395 (3.294–3.5)	1.502 (1.436–1.569)	

**A**: Pairwise *F*_*st*_, *G’*_*st*_, and Jost’s *D* values (above diagonal; 1^st^, 2^nd^ and 3^rd^ rows, respectively) and pairwise CSE distances (below diagonal) are given together with significance levels (* < 0.005; ** <0.0005) based on Bonferroni corrections. **B**: Number of migrating individuals per generation between species, as estimated by MIGRATE– 95% c.i. bounds are in parenthesis; values in first and second rows are based on 12 and 8 loci, respectively; columns indicate source species and rows indicate receiving species.

Our initial likelihood analysis of “first generation migrants” using GENECLASS indicated that one phenotypical *L*. *starcki* neonate collected in the Abijata-Shala National Park in the Rift Valley (AB, Fig 1) has been misidentified, in fact belonging to *L*. *habessinicus* (likelihoods: *L*. *starcki* = -37.08; *L*. *habessinicus* = -16.367; *L*. *fagani* = -28.931). After allocating that specimen to *L*. *habessinicus*, Bayesian species assignments of individuals matched in all cases the phenotypically determined species, when based on 12 or occasionally slightly fewer loci (due to missing genotypes); however, one (phenotypic and molecular) *L*. *habessinicus* was assigned to *L*. *fagani* when using only eight loci.

The NJ tree (S1 Fig) based on pairwise individual CSE distances revealed a set of seven minor or more comprehensive clusters of *L*. *habessinicus* individuals very closely related to each other, one distinct *L*. *fagani* clade, closely related to two of the *L*. *habessinicus* clades, and a distinct *L*. *starcki* clade, slightly more separate from the *L*. *habessinicus* clades and most distant from the *L*. *fagani* clade. Two *L*. *starcki* and two *L*. *fagani* individuals clustered within three *L*. *habessinicus* clades and three *L*. *habessinicus* individuals clustered within the *L*. *fagani* clade. However, bootstrap support was indicated only for the *L*. *starcki* clade (78%), thereby confirming the two phenotypical *L*. *starcki* clustering among *L*. *habessinicus* individuals being closer related to the latter.

**Fig 6:**
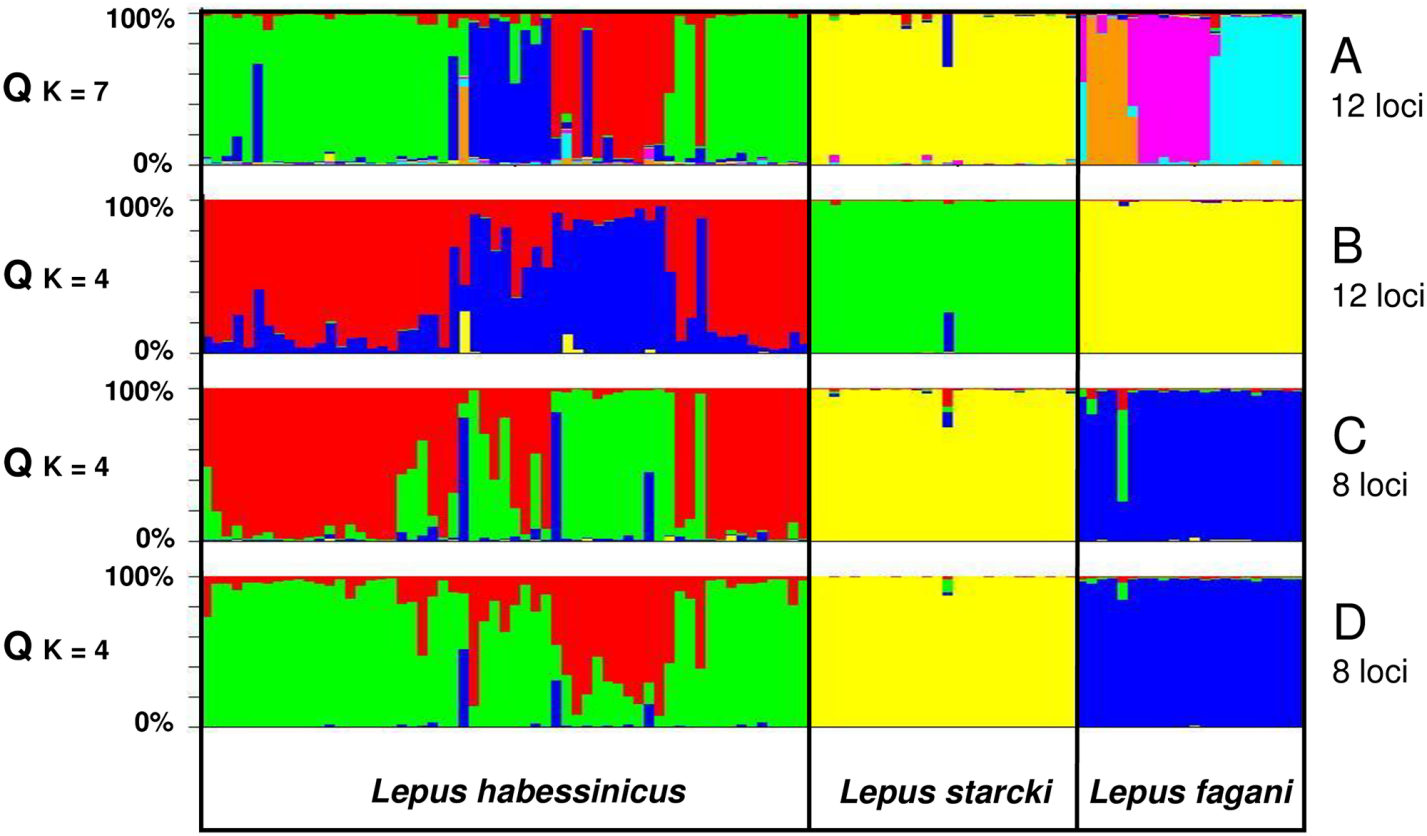
Microsatellite-based Bayesian structure and admixture analysis of the genotypes of the three Ethiopian hare species. Model results are based on A: 12 loci, correlated allele frequencies, and no species priors, B: 12 loci, correlated allele frequencies, species priors, C: 8 loci, correlated allele frequencies, no species priors, D: 8 loci, correlated allele frequencies, species priors. For more details see “Material and methods”.

STRUCTURE (Fig 6) revealed seven most likely genetic clusters inherent to the total data set (12 loci) for the runs without prior species information (mean/s.d. of Ln P(D), K = 6: -3413.0/11.5; K = 7: -3374.9/3.2; K = 8: -3416.7/49.0). For runs with prior species (phenotype) information the most likely result was K = 4 (mean/s.d. of Ln P(D); K = 3: -3629.7/ 3.3; K = 4: -3511.7/4.1; K = 5: -3487.5/33.9). For admixture models with eight loci K = 4 were the most likely results both for the runs without (mean/s.d. of LnP(D); K = 3: -2399.0/0.3; K = 4: -2331.4/0.4; K = 5: -2335.8/40,5) and with “species” as prior (K = 3: -2405.2/3.04; K = 4: -2343.5/6.2; K = 5: -2387.6/13.2). In all runs, some individuals consistently featured relatively high proportions (Q) of clusters typical for other species, respectively: for the set of 12 loci (no “species” prior), the phenotypical and molecular *L*. *habessinicus* individual #26 featured *L*. *fagani*-type clusters at Q = 58.3%, which was above the six-fold upper bound of the standard deviation (6 x s.d. = 50.99%) of individual Q values for *L*. *fagani*-type clusters in 59 *L*. *habessinicus* individuals; similarly, *L*. *starcki* #73 featured a *L*. *habessinicus*-type cluster at Q = 34.5%, well above the four-fold upper bound of the s.d. (= 28.53%) of Q values for *L*. *habessinicus* in *L*. *starcki* hares. Thus, we considered those latter two individuals as recent hybrids, rather than as drift signals of ancestral alleles. A few more hares might be viewed as offspring of recent hybrids, but the admixture results were not concordant in all STRUCTURE models. Our panel of eight loci, however, did not fully qualify for identifying modern hybrids. Notably, our STRUCTURE models suggested significant substructures within *L*. *habessinicus* and *L*. *fagani* without much admixture, respectively.

Current gene flow between species as estimated by MIGRATE ranged between 0.311 and 2.447 individuals per generation for the 12 loci data set and between 0.265 and 3.360 individuals per generation for the 8 loci data set, respectively (Table 3). Whereas migration was rather balanced between *L*. *habessinicus* and *L*. *starcki*, it was asymmetric between *L*. *habessinicus* and *L*. *fagani*, and between *L*. *starcki* and *L*. *fagani*, with clearly less gene flow from *L*. *fagani* to either species.

## Discussion

### Phenotypes and molecular species assignment

Given the possibility of regionally varying scenarios of reticulate evolution (e.g., Thulin and Tegelström[119], Alves et al.[8], Liu et al.[9], Melo-Ferreira et al.[10,11]) and the large phenotypic variance within and among species or taxa Angermann [2], molecular phylogenetic studies of the genus *Lepus* must be accompanied at least by external phenotype analysis in regions of species sympatry or parapatry. This should also help to prevent ambiguities and future confusions of molecular data archived in repositories.

All presently collected hares from Ethiopia could be unambiguously assigned to Ethiopian hares *Lepus fagani*, Abyssinian hares, *L*. *habessinicus*, or Ethiopian Highland hares, *L*. *starcki*, based on their external phenotype, except for one subadult individual from Assosa (AS), western Ethiopia, close to the North Sudan border, and one neonate *L*. *habessinicus* from the Abijata-Shalla National Park (AB) in the central Ethiopian Rift Valley (Fig 1). The remains of the external phenotype and the ATP6 haplotype of the former (from AS) corresponded by and large to that of typical *L*. *fagani*, but due to technical reasons we repeatedly failed to obtain meaningful microsatellite data for its final molecular assignment. The neonate from AB, however, featured an external phenotype typical for *L*. *starcki*, but was undoubtedly assigned to *L*. *habessinicus* by all our molecular markers. That mismatch was not due to possible sample confusion, because that individual featured a unique multi-locus microsatellite profile, when cross-checked for its relationship with all other *L*. *habessinicus* individuals by Queller and Goodnight [120] r_xy_ indices (using IDENTIX 1.0/1.0.2 [121]). Moreover, no hint of possible introgression by *L*. *starcki* was detected in that neonate, as revealed by allele/genotype comparisons, STRUCTURE, and the Bayesian assignment based on its full microsatellite set. Generally, within *Lepus* phenotype-based species determination of neonates is deemed impossible, though–as to our knowledge–not systematically studied. However, the conspicuous outer pinnae pattern and the often entire white upper surface of the tail represent a combination of external characteristics of *L*. *starcki* that cannot be confused with phenotypes of other hares occurring in Ethiopia (e.g., Yalden et al.[60]). Notably, the single neonate with a *L*. *starcki* phenotype from the Abijata-Shalla National Park in the Rift Valley did display those two characteristics very distinctly. No specific results are available to interpret that phenotype/molecular data mismatch. Possibly, few introgressed genes underlying the addressed external characteristics (e.g., coat colour genes; Koutsogiannouli et al.[122]) were not reflected by our overall microsatellite set. Notably, despite distinct coat colour differences, only shallow molecular divergence was observed among North African cape hares (*L*. *capensis*) and hares from Israel, cf., *L*. *capensis* and *L*. *europaeus* Ben Slimen et al.[21,37], as well as between Mandshurian hares (*L*. *mandshuricus*) and the blackish “*L*. *melainus*” form from eastern Asia considered conspecific with *L*. *mandshuricus* [44,123]. Even certain alleles of single coat colour genes (*TYR*, *MC1R*) did not predict the two winter coat types in Japanese hares, *L*. *brachyurus* [45].

The vast majority (99.1%) of all our comparisons between phenotype and molecular data, nevertheless, resulted in unambiguous species determination by external phenotypes alone, without doubtful intermediate forms hinting towards possible hybrids. External phenotypes of Somalian cape hares, *L*. *capensis*, as determined by Azzaroli-Puccetti[57], however, are very similar to our *L*. *habessinicus* (unpub. data of one of the authors (FS) collected at the Museum of Zoology and Natural History, “La Specola”, Florence, Italy). Moreover, several skins in the collection of “La Specola” (Florence) assigned to *L*. *crawshayi* (= *L*. *victoriae*) by M. L. Azzaroli-Puzzetti are virtually indistinguishable from standard “*L*. *habessinicus*” phenotypes (pers. observ. of FS). Similarly, external phenotypes of the currently investigated *L*. *fagani* may not differ much from those of some forms of East African savanna hares, *L*. *victoriae* (e.g., Angermann[1], Flux and Flux[117], see also Yalden et al.[60], Flux and Angermann[5]). All these ambiguities call for thorough examination of phenotypic character variation, including skull and dental characters as well (e.g., Palacios et al.[39]) in the context of molecular phylogenetic studies of hares (from Africa).

### Molecular phylogenetic relationships among hares from Ethiopia and other closely related species

Somewhat unexpectedly, both our ATP6 and TF sequences indicate generally very close phylogenetic relationships between *L*. *fagani*, *L*. *habessinicus*, and *L*. *starcki*, despite their clear phenotypic and genetic distinction as reflected by microsatellites. All three species are paraphyletic both in their mitochondrial and nuclear sequences, which is not uncommon in the genus *Lepus* (e.g., Alves et al.[8], Wu et al.[19], Melo-Ferreira et al.[18], Ben Slimen et al.[21], Melo-Ferreira et al.[22], Liu et al.[9]). Particularly during short periods of adaptive radiation and with large effective sizes of ancestral populations incomplete lineage sorting is likely to result in shared ancestral polymorphism in modern species (e.g., Degnan and Rosenberg[124]). Although modern hares and jackrabbits (*Lepus*) may represent evolutionarily young offshoots of (now extinct) forms given currently genera ranks in paleontological taxonomy, likely dating back to the late Miocene (see e.g., Matthee et al.[125]), their major and rapid adaptive radiation has happened only in the recent past, as inferred from paleontological (e.g., Lopes-Martinez[126]) and molecular studies (Robinson and Matthee[127], and references therein); supposed pre-Pliocene *Lepus* finds in Africa, need a thorough revision [128]. Effective sizes of ancestral hare populations with little ecological adaptation to different niches and little behavioural specifications may have been large, especially at shallow genetic divergence across large ranges (comp. Campos et al.[129] for Ne estimates of extant *L*. *europaeus*) and this might have favored incomplete lineage sorting. Regarding potential incomplete lineage sorting of the currently studied *MT-ATP6* sequences would not exclude (temporal and spatial) scenarios of positive or negative selection[10].

### Relationships between *Lepus habessinicus* and *L*. *capensis*

Our mtATP6 data (Figs 2 and 3) indicate that *L*. *habessinicus* and the other two species from Ethiopia are clearly evolutionarily separated from South and North African *L*. *capensis*, without any hint of shared ancestral polymorphism. Thus, for *L*. *habessinicus*, the straightforward evolutionary interpretation based on mtDNA would suggest a species quite distinct from *L*. *capensis*, in agreement with Azzaroli-Puccetti [57,58]. On the other hand, in our evolutionary network model the most ancestral haplotype of *L*. *habessinicus* holds a position close to where the lineages of *L*. *europaeus*, *L*. *timidus*, and *L*. *capensis* meet and close to the *L*. *europaeus* phylogroup. This may suggest its relatively ancestral origin together with *L*. *europaeus*. Notably, one haplotype of *L*. *saxatilis* (scrub hare) is ancestral to both *L*. *habessinicus* and *L*. *europaeus* and shares one relatively old haplotype with North African *L*. *capensis* and *L*. *europaeus*, though the latter species does not occur in Africa, and *L*. *saxatilis* occurs only in South Africa, where it does obviously not share haplotypes with the sympatric *L*. *capensis*. The most ancestral *L*. *habessinicus* haplotype is also shared by *L*. *saxatilis*. This scenario might suggest that lineages that are currently found in *L*. *saxatilis*, *L*. *europaeus*, the ancestral *L*. *habessinicus* phylogroup, and occasionally in North African *L*. *capensis* are amongst the most ancestral of all currently studied species. Thus, alternative to the straightforward phylogenetic interpretation above, we hypothesize that *L*. *habessinicus* represents a comparatively old descendant of an “ancestral *L*. *capensis* form” (a precursor of modern *L*. *capensis* sensu lato), probably originating from eastern or central Africa, that has given rise to lineages now found in modern South and North African *L*. *capensis*. This hypothesis is supported by our transferrin (TF) sequences: 1) they indicate a close evolutionary relationship between South and North African cape hares and 2) all African *L*. *capensis* haplotypes are descendants of the most ancestral *L*. *habessinicus* haplotype that is also the most ancestral among all haplotypes found in the currently studied African taxa and all other *Lepus* species from America and Eurasia, except for one haplotype of *L*. *californicus* from America and one of *L*. *hainanus* from Southeast Asia (Fig 4); the latter two haplotypes link to the outgroup genera *Oryctolagus* and *Sylvilagus*.

Our hypothesis of *L*. *habessinicus* representing a close taxon to an “ancestral *L*. *capensis* form” would help to explain the close phenotypic and morphological relationships among *L*. *habessinicus* and all South and North African forms of *L*. *capensis* (“*L*. *capensis* sensu lato”: Angermann[1,2], Yalden et al.[60], Flux and Angermann[5], see also Azzaroli-Puzzetti[57,58]). It could further explain the big divergence of mitochondrial control region sequences between South and North African cape hares Suchentrunk et al.[6], despite their clearly lower mtDNA divergence as estimated by RFLPs Ben Slimen et al.[52], and their quite close genetic relationship as estimated by allozymes Suchentrunk et al.[6] and microsatellites Ben Slimen et al.[37]. Obviously, mitochondrial control region sequences can reach remarkably high divergence levels in hares with close nuclear gene pool relationships, as in South and North African cape hares Kryger[55], Ben Slimen et al.[31], Suchentrunk et al.[40,41].

Mitochondrial and nuclear sequences of phenotypical East African cape hares (e.g., Flux and Flux[117]) should allow testing our hypothesis of the ancestral status of *L*. *habessinicus* and its close relationship to East African *L*. *capensis* connecting cape hares from South and North Africa. Results based on few available mtDNA and nuclear sequences of African *L*. *capensis* [130, 33, 8,125, 21], however, are ambiguous. For instance, European brown hares (*L*. *europaeus*), Iberian hares (*L*. *granatensis*), and several American *Lepus* species appear ancestral to (North) African cape hares in cyt b sequences [8]. On the contrary, mitochondrial control region sequences of South African cape hares are basal to all other taxa studied, whereas control region sequences of North African cape hares are closely related to those of *L*. *europaeus* and *L*. *saxatilis* Ben Slimen et al.[21]; however, in all those analyses basal nodes were not supported, and different sets of taxa were used for the different phylogeny models.

Our TF sequence results indicate that *L*. *europaeus* appears evolutionarily younger than all African *L*. *capensis* and *L*. *habessinicus*, whereas South African scrub hares, *L*. *saxatilis*, may hold a relatively ancestral position. The evolutionary relationship between South African *L*. *saxatilis* and African savanna hares, *L*. *victoriae*, however, is not fully understood. Microsatellite data Kryger[55] indicate high gene flow between *L*. *saxatilis* from SW South Africa and the range of *L*. *victoriae* in N and NE South Africa, Botswana, Namibia, and parts of Zimbawe, where *L*. *saxatilis* has traditionally been considered absent. A conspecific status of those two taxa as proposed by Robinson and Dippenaar [51] (see also Collins[50]), for instance with clinal genetic divergence (i.e., isolation by distance) and a resultant species range from South to North Africa of *L*. *saxatilis* (being hypothetically conspecific with *L*. *victoriae*) could significantly influence the evolutionary interpretation of the currently revealed ATP6 haplotypes shared by *L*. *saxatilis* and hares from Ethiopia. The generally not well supported basal nodes of mtDNA-based phylogenies of the genus *Lepus* published so far are congruent with a supposed quick radiation of the genus *Lepus* during the Pleistocene (e.g., Matthee et al.[125]). This calls for numerous samples from geographically dispersed origins and comprehensive molecular marker sets to unravel detailed phylogenetic relationships of African hares.

#### Phylogenetic position of *Lepus starcki*

The Ethiopian highland hare, *Lepus starcki*, has traditionally been viewed as closely related to *L*. *capensis* or *L*. *europaeus*, or possibly representing a relict form of one or both of them (e.g., Petter[59], Angermann[1]; see also Yalden et al.[60,61], Azzaroli-Puccetti[57,58], Flux and Angermann[5]). Phenotypically, it is very distinct from all other East African species; its external phenotype superficially resembles that of *L*. *europaeus*, a species absent from Africa. All but one mitochondrial lineages of *L*. *starcki* occur in a derived haplogroup (“*L*. *starcki* haplogroup”; see Fig 2), related to the modern haplogroup of *L*. *habessinicus;* this suggests its rather recent evolution from *L*. *habessinicus*. The two mitochondrial *L*. *starcki* and *L*. *habessinicus* sequences published by Pierpaoli et al. [33], our microsatellite and TF sequences results are in principle not contradictory to that interpretation. The presence of three haplotypes of the “*L*. *starcki* haplogroup” in individuals of *L*. *habessinicus* does agree with this interpretation as well, and the occurrence of a single *L*. *starcki* haplotype in the ancestral *L*. *habessinicus* haplogroup may be viewed as shared ancestral polymorphism, or ancient or modern introgression.

However, *L*. *starcki* is the only African hare species that harbors haplotypes in all three (ancestral and modern) major TF phylogroups. Notably, it shares one TF haplotype with the Holarctic *L*. *arcticus*, *L*. *othus*, *L*. *timidus* (i.e., *L*. *timidus* complex) and with *L*. *townsendii*. On the contrary, one *L*. *timidus* individual shares the most ancestral haplotype among all African species with *L*. *starcki*, *L*. *habessinicus*, North African cape hares, and one central Asiatic specimen of *L*. *yarkandensis*. *Lepus arcticus*, *L*. *othus*, *L*. *timidus*, and *L*. *townsendii* are all closely related forms (e.g., Flux and Angermann[5], Melo-Ferreira et al.[28]) absent from Africa, but adapted to cold Palearctic or Nearctic climate regions in the Palearctic or Nearctic. Taking into consideration the results of all three currently studied molecular marker systems, the most parsimonious phylogenetic interpretation would be that *L*. *starcki* has acquired that relatively modern TF haplotype shared with the cold adapted Palearctic and Nearctic hares by ancient introgression during an early phase of adaptive radiation starting from a (Holarctic?) precursor form that has led to the modern *L*. *timidus* complex (and to *L*. *townsendii*). This could have happened during one of the cold phases of the middle or late Pleistocene under a range expansion of either *L*. *starcki* or the (hypothetical) Holarctic precursor, or of both; it would help to explain the adaptation of *L*. *starcki* to cooler environments in tropical East Africa, i.e., its restriction to the Ethiopian highlands, east and west of the Rift Valley (e.g., Yalden et al.[60], Flux and Angermann[5]). The surface of the tail is often fully white in *L*. *starcki* [60,5], in congruence with many forms of the *L*. *timidus* complex and *L*. *townsendii*; and that might hint towards such a hypothesized ancient introgression by an arcto-alpine precursor of the modern *L*. *timidus* complex and *L*. *townsendii*.

An alternative interpretation could be that *L*. *starcki* represents an ancestral (relict) species that has relatively recently been massively introgressed by expanding *L*. *habessinicus*, as observed for mtDNA of Chinese *L*. *mandshuricus* that has been entirely replaced by that of *L*. *timidus* or *L*. *sinensis* [9]. Our microsatellite data, however, do not readily support this interpretation, though the significant signal of a reduction of its effective population size in the more distant past is not incongruent with such an evolutionary scenario. Future more geographically spaced *L*. *starcki* and *L*. *timidus* samples, together with multiple nuclear sequences, should enable evaluating the likelihood and extent of ancient introgression of ancestral *L*. *timidus* and allied cold-adapted taxa into *L*. *starcki*, similar to the scenario revealed in hares from the Iberian Peninsula (e.g., Alves et al.[8], Melo-Ferreira et al.[28]) or the massive introgression of *L*. *timidus* into Chinese *L*. *capensis* [9].

Another potential interpretation could be that the TF sequence shared by *L*. *starcki* and forms of the *L*. *timidus* complex and *L*. *townsendii* represents in fact a *L*. *habessinicus* haplotype that has given rise to the haplotypes present in the modern *L*. *timidus* complex and *L*. *townsendii*, as well as in *L*. *starcki*, but has so far not been detected in *L*. *habessinicus* itself, due e.g., to our restricted geographical sampling. The one *L*. *timidus* individual that shares the most ancestral transferrin haplotype of all *L*. *habessinicus* haplotypes lends support for this hypothesis, but without comprehensive geographical TF data for both *L*. *habessinicus* and *L*. *starcki* we consider it rather speculative.

Still more evolutionary scenarios are conceivable for *L*. *starcki*, such as ancient introgression by *L*. *europaeus* that has in turn been introgressed by *L*. *timidus*; the 1bp-long transferrin insertion shared by *L*. *starcki*, *L*. *europaeus*, and *L*. *timidus*, as well as similar external phenotypes, skulls, and dental characters of *L*. *starcki* and *L*. *europaeus* might suggest this. However, a thorough evaluation of such alternative hypotheses needs a much more comprehensive arrangement of samples and molecular makers.

#### *Lepus fagani*—Young or ancestral species?

The Ethiopian hare, *Lepus fagani*, represents one of the least known *Lepus* forms currently given species rank (e.g, Flux and Angermann[5], Hoffmann and Smith[12], Alves and Hackländer[13]). As to our knowledge no molecular data have so far been published on it. Equally, no modern literature on phenotypes and morphometry or other aspects of biology does exist for that taxon, nor is its range reasonably described. Traditionally, it is considered either a subspecies of the African savanna hare, *L*. *victoriae*, or as a separate species closely related to the latter based on earlier descriptions of fur, skull, and dental characteristics (e.g., Angermann[1], Yalden et al.[60,61], Azzaroli-Puccetti[57,58], Flux and Angermann[5]).

Our microsatellite results and TF sequences concordantly indicate a very young evolutionary descent of *L*. *fagani* from *L*. *habessinicus*, strikingly contradicting the traditional systematic view. The mitochondrial ATP6 sequences are also not incongruent with the hypothesis of a very recent evolution from the most basal *L*. *habessinicus* haplotype or with the hypothesis of shared ancestral polymorphism and relatively recent or ancestral introgression by lineages of the (more derived) haplogroup in hares from Ethiopia that is otherwise typical for *L*. *starcki*. Alternatively, the ATP6 network of *L*. *fagani* haplotypes may be interpreted as representing an ancestral lineage system that has totally been substituted by *L*. *habessinicus* lineages and the “*L*. *starcki* haplogroup” (i.e., “mitochondrial capture”). In fact, a “*L*. *fagani*-typical ATP6 haplogroup” or even haplotype does not exist, and one haplotype that occurs exclusively in one single *L*. *fagani* individual holds an ancestral position equal to the most ancestral *L*. *habessinicus* haplotype; those findings are not incongruent with the hypothesis of an ancestral position of *L*. *fagani* and its mitochondrial capture by *L*. *habessinicus*/*L*. *starcki*. Nevertheless, we consider this hypothesis as less likely, because neither microsatellites nor the TF sequences suggest ancestral states compared to *L*. *habessinicus*: TF haplotypes of *L*. *fagani* are all younger than the most ancestral *L*. *habessinicus* haplotype, and multilocus microsatellite data indicate a very recent differentiation of *L*. *fagani* compared to a somewhat earlier differentiation of *L*. *starcki* from *L*. *habessinicus* (in accordance with ATP6 sequences). Given the ancestral bottleneck signal for *L*. *starcki*, however, the multilocus genetic divergence between *L*. *starcki* and *L*. *habessinicus* might have been somewhat inflated due to a possible drift effect in the former species. The TF sequences suggest a rather similar recent evolutionary divergence of either species from *L*. *habessinicus*. As 33 of all found microsatellite alleles are autapomorhic to *L*. *fagani*, the hypothesis of full mitochondrial and nuclear substitution of an ancestral *L*. *fagani* species by *L*. *habessinicus* would mean that 22.6% of all currently found alleles may in fact represent modern or ancestral *L*. *habessinicus* alleles, but do not any longer exist in modern *L*. *habessinicus* (or have at least not been found currently); that assumption seems, however, fairly unlikely. The allelic contribution of other African hare species (*L*. *victoriae*; *L*. *saxatilis*) in earlier phases of evolution to *L*. *fagani* is unknown; however, a late Pleistocene distribution of *L*. *saxatilis* across large parts of Africa and at least regional admixture with *L*. *victoriae* is conceivable, and such a scenario might have provided gene pool sources for *L*. *fagani*. This would explain the phenotypic similarity of *L*. *fagani* with *L*. *victoriae* (e.g., Angermann[1], Yalden et al.[60], Flux and Angermann[5]) that is considered closely related to or even conspecific with *L*. *saxatilis*. The hypothesis of a hybridogenic origin of *L*. *fagani* can only be explored by including many *L*. *victoriae* / *L*. *saxatilis* data into the analysis.

### Contemporaneous introgressive hybridization in hares from Ethiopia

Already Halanych et al.[130] noticed that *Lepus* species that are known to hybridize in the wild, such as *L*. *europaeus* and *L*. *timidus*, may nevertheless show marked mtDNA divergence, indicative of “good species”. He hypothesized that geographic, ecological, or behavioural isolation mechanisms may be driving speciation in the genus *Lepus* rather than genetic incompatibility, also in view of the very little chromosomal variation within the genus (e.g., Robinson et al.[131,132], Azzaroli-Puccetti et al.[133]). Later studies are not incongruent with this hypothesis (e.g., Alves et al.[8], Liu et al.[9], Wu et al.[27], Melo-Ferreira et al.[10,11], see also Thulin et al.[7], and Robinson and Matthee[127]).

Indeed, our microsatellite admixture results revealed two cases (1.9%) of contemporaneous introgressive hybridization in the hares from Ethiopia, albeit external phenotypes did not suggest hybrids. One nuclear genome of a *L*. *habessinicus* individual was introgressed by *L*. *fagani* and one nuclear genome of a *L*. *starcki* individual was introgressed by *L*. *habessinicus*. A few more hares might have been introgressed recently in their nuclear genomes, but inconsistent Bayesian admixture models did not confirm that. The absence of intermediate phenotypes conforms to the relatively rare occurrence of current nuclear introgressive hybridization. However, given that only a relatively small proportion (25%) of hares were collected from sympatric areas, introgressive hybridization might actually occur at somewhat higher species-wide levels, respectively. That is suggested by our coalescence theory-based gene flow results, both for the full and the reduced set of loci: for *L*. *habessinicus* and *L*. *starcki* estimated numbers of individuals migrating per generation are all above 1.0; theoretically, that level of migration is sufficient to compensate genetic drift or reduce interspecific divergence between species/populations under an island population model [134]. For *L*. *fagani*, however, we observed asymmetric gene exchange with *L*. *habessinicus* and *L*. *starcki*, with clearly less than one *L*. *fagani* individual per generation introgressing into the other two species, respectively. Nevertheless, we observed one *L*. *habessinicus* with its nuclear DNA having been introgressed in one of its recent ancestors by *L*. *fagani*. However, all these migration estimates may vary according to the geographical distribution of specimen samples. i.e., the proportion of hares collected sympatrically, pararapatrically, or allopatrically. Hence, a more developed discussion of geographic variation of nuclear introgression frequencies requires geographically more comprehensive data. Overall, ongoing nuclear DNA introgression seems to occur at low level, possibly similar to that found between *L*. *granatensis* and *L*. *europaeus* in northern Iberia Melo-Ferreira et al.[11] and *L*.*europaeus* and *L*. *timidus* [17,26].

### Conclusions and systematic remarks

The present study represents the first comprehensive molecular phylogenetic investigation of hares (*Lepus*) from Ethiopia. It reveals a complex molecular evolutionary scenario: both mitochondrial and nuclear DNA exhibit shared ancestral polymorphism among hares from Ethiopia and other species from Africa and Europe. Moreover, nuclear TF sequences indicate shared ancestral polymorphism among African, Eurasian, and American hare species (see also Awadi et al. [135]). Both our microsatellite and mtDNA data indicate very close evolutionary relationships of all three species collected from Ethiopia, the Abyssinian hare, *L*. *habessinicus*, the Ethiopian hare, *L*. *fagani*, and the Ethiopian Highland hare, *L*. *starcki*, despite their very good phenotypic distinction, without intermediate forms that might suggest recent hybridization. Nevertheless, occasional hybridization does occur in hares from Ethiopia, possibly at somewhat higher frequencies in areas of sympatry, as suggested by our coalescence theory-based gene flow results. Unexpectedly, and in strong contradiction to traditional taxonomy based on phenotypic and morphological characters, all our molecular markers indicate recent descent of *L*. *fagani* from *L*. *habessinicus*. Almost complete mitochondrial substitution of (an ancestral form of) *L*. *fagani* by *L*. *habessinicus* is not inconsistent with our molecular data, but is deemed unlikely for nuclear DNA. Massive ancient introgression of ancestral forms of modern African savanna hares, *L*. *victoriae*, and/or scrub hares, *L*. *saxatilis*, into parts of an ancestral form of *L*. *habessinicus* with secondary separate evolution into modern *L*. *fagani* might represent an additional alternative evolutionary hypothesis for *L*. *fagani*, but remains to be tested by many geographically meaningful samples and additional molecular markers. Also unexpected is the evolutionary position of *L*. *starcki* close to *L*. *habessinicus*. It might have received ancient gene flow from currently allopatric *L*. *timidus* and/or *L*. *europaeus*, possibly during periods of range expansion of those species. The inclusion of many phenotypical *L*. *capensis* sensu lato from eastern Africa should help to understand the relationship between *L*. *habessinicus* and *L*. *capensis* sensu lato. Despite the complicated reticulate evolutionary scenario, ongoing gene exchange, their close evolutionary relationships, and pending data on evolutionary relationships particularly of cape hares, *L*. *capensis* sensu lato, and forms of the *L*. *saxatilis*/*L*. *victoriae* complex, our results are not incongruent with the currently acknowledged separate species status of *L*. *fagani*, *L*. *habessinicus*, and *L*. *starcki* from Ethiopia. This conclusion conforms to the current *Lepus* taxonomy (e.g., Alves and Hackländer[13]), and to a “relaxed biological species definition” (e.g., Coyne and Orr [136]) that allows for some gene flow between species, provided main evolutionary trajectories for species are not corrupted.

## Supporting information

S1 Fig NJ dendrogram of individual hares based on pairwise Cavalli-Sforza & Edwards distances as obtained from twelve microsatellite loci. Bootstrap support is shown, if above 50%. For details see “Material and methods”.
(TIF)

S1 Table Accession numbers of the downloaded transferrrin (TF; n = 111; i.e. from NCBI) sequences. (DOC)

S2 Table Accession numbers, frequencies, and taxon names of the ATP6 sequences produced in this study; haplotype numbers per taxon are given in parentheses. (DOC)

S3 Table Net mean between group (i.e., species) p distances of ATP6 sequences. (DOC)

S4 Table Accession numbers, frequencies, and taxon names of the phased TF sequences produced in this study; haplotype numbers per taxon are given in parentheses. (DOC)

S5 Table Pairwise p-distances of Transferrin sequences. CS–*L*. *capensis*, South Africa, CN–*L*. *capensis*, North Africa, E–*L*. *europaeus*, F–*L*. *fagani*, H–*L*. *habessinicus*, X–*L*. *saxatilis*, S–*L*. *starcki*, T–*L*. *timidus*, M–*L*. *mandshuricus*, TW–*L*. *townsendii*, CF–*L*. *californicus*, AM–*L*. *americanus*, OT–*L*. *othus*, AR–*L*. *arcticus*, CC–*L*. *capensis*, China, C–*L*. *corsicanus*, G–*L*. *granatensis*, OI–*L*. *oiostolus*, CO–*L*. *comus*, Y–*L*. *yarkandensis*, HN–*L*. *hainanus*, SI–*L*. *sinensis*, CJ–*L*. *castroviejoi*. Minimum and maximum are in red, respectively.
(DOC)

## References

[1] Angermann R,. Revision der paläarktischen und äthiopischen Arten der Gattung *Lepus* (Leporidae, Lagomorpha). Diss. Thesis, Humboldt University of Berlin.1965

[2] Angermann R,. The taxononmy of Old World *Lepus*. Acta Zool. Fennica. 1983; 174, 17–21.

[3] Palacios F,. Biometric and morphologic features of the species of the genus *Lepus* in Spain. Mammalia. 1989;73, 227–264.

[4] Palacios F,. Systematics of the indigenous hares of Italy traditionally identified as Lepus europaeus Pallas, 1778 (Mammalia: Leporidae). Bonn. Zool. Beitr. 1996; 46, 59–91.

[5] Flux JEC, Angemann R. The hares and jackrabbits, in: Chapmann JA, Flux JEC (Eds.), Rabbits, Hares and Pikas: status conservation Action Plan. IUCN resources Gland, Swiitzerland, 1990; pp. 61–94.

[6] Suchentrunk F, Ben Slimen H, Sert H,. Phylogenetic Aspects of Nuclear and Mitochondrial Gene-Pool Characteristics of South and North African Cape Hares (Lepus capensis) and European Hares (*Lepus europaeus*), in: Alves PC, Ferrand N, Hackländer K (Eds), Lagomorph Biology: Evolution, Ecology and Conservation. Springer, Berlin, Heidelberg, 2008; pp. 65–88.

[7] Thulin CG, Jaarola M, Tegelström H. The occurrence of mountain hare mitochondrial DNA in wild brown hares. Mol. Ecol. 1997; 6, 463–467. 9161014 10.1046/j.1365-294x.1997.t01-1-00199.x

[8] Alves PC, Ferrand N, Suchentrunk F, Harris DJ. Ancient introgression of *Lepus timidus* mtDNA into *L*. *granatensis* and *L*. *europaeus* in the Iberian Peninsula. Mol. Phylogent. Evol. 2003; 27, 70–80. 10.1016/s1055-7903(02)00417-7 12679072

[9] Liu J, Yu L, Arnold ML, Wu CH, Wu SF, Lu X et al Reticulate evolution: frequent introgressive hybridization among Chinese hares (genus Lepus) revealed by analysis of multiple mitochondrial and nuclear DNA loci. BMC Evol. Biol. 2011b; 11, 223–236. 21794180 10.1186/1471-2148-11-223 PMC3155923

[10] Melo-Ferreira J, Vilela J, Fonseca MM, da Fonseca RR, Boursot F, Alves PC. The elusive nature of adaptive mitochondrial DNA evolution of an arctic lineage prone to frequent introgression. Genome Biol. Evol. 2014a; 6, 886–896. 24696399 10.1093/gbe/evu059 PMC4007550

[11] Melo-Ferreira J, Seixas FA, Cheng E, Mills SL, Alves PC,. The hidden history of the snowshoe hare, *Lepus americanus*: extensive mitochondrial DNA introgression inferred from multilocus genetic variation. Mol. Ecol. 2014b; 23, 4617–4630. 25113393 10.1111/mec.12886

[12] Hoffmann RS, Smith AT, Order Lagomorpha, in: Wilson DE, Reeder DAM (Eds.), Mammal Species of the World, 3rd ed., vol.1, J. Hopkins Univ. Press, Baltimore, 2005; pp. 185–211.

[13] Alves PC, Hackländer K. Lagomorph Species: Geographical Distribution and Conservation Status, in: Alves PC, Ferrand N, Hackländer K (Eds.), Lagomorph Biology: Evolution, Ecology, and Conservation. Springer, Berlin, Heidelberg, 2008; pp. 395–405.

[14] Kasapids P, Suchentrunk F, Magoulas A, Kotoulas G, The shaping of mitochondrial DNA phylogeographic patterns of brown hare (*Lepus europaeus*) under the combined influence of Late Pleistocene climatic fluctuations and Anthropogenic translocations. Mol. Phylogent. Evol. 2005; 34, 55–66. 10.1016/j.ympev.2004.09.007 15579381

[15] Stamatis C, Suchentrunk F, Moutou KA, Giacometti M, Haerer G, Djan M, et al Phylogeography of the brown hare (Lepus europaeus) in Europe: a legacy of south–eastern Mediterranean refugia? J. Biogeogr. 2009; 36, 515–528.

[16] Alves PC, Harris DJ, Melo-Ferreira J, Branco M, Suchentrunk F, Boursot P, et al Hares on thin ice: Introgression of mitochondrial DNA in the hares and its implications for recent phylogenetic analyses. Mol. Phylogent. Evol. 2006; 40, 640–641. 10.1016/j.ympev.2006.02.016 16624594

[17] Thulin CG, Fang M, Averianov A O,. Introgression from *Lepus europaeus* to *L*. *timidus* in Russia revealed by mitochondrial single nucleotide polymorphism and nuclear microsatellite. Hereditas. 2006a; 143, 68–76. doi: 10.1111/j.2006.0018-0661.01952.x 17362337 10.1111/j.2006.0018-0661.01952.x

[18] Melo-Ferreira J, Boursot P, Suchentrunk F, Ferrand N, Alves PC, Invasion from the cold past: extensive introgression of mountain hare (*Lepus timidus*) mitochondrial DNA into three other hare species in northern Iberia. Mol. Ecol. 2005; 14, 2459–2464. doi: 10.1111/j.1365-294X.2005.02599.x 15969727 10.1111/j.1365-294X.2005.02599.x

[19] Wu C, Wu J, Bunch TD, Li Q, Wang Y, Zhang YP Molecular phylogenetics and biogeography of Lepus in Eastern Asia based on mitochondrial DNA sequences. Mol. Phylogent. Evol. 2005; 37, 45–61. 10.1016/j.ympev.2005.05.006 15990340

[20] Fredsted T, Wincentz T, Villesen P. Introgression of mountain hare (*Lepus timidus*) mitochondrial DNA into wild brown hares (*Lepus europaeus*) in Denmark. BMC Ecol. 2006; 6, 17 doi: 10.1186/1472-6785-6-17 17105672 10.1186/1472-6785-6-17 PMC1654140

[21] Ben Slimen H, Suchentrunk F, Shahin AB, Ben Ammar Elgaaied A.. Phylogenetic analysis of mtCR-1 sequences of Tunisian and Egyptian hares (*Lepus* sp. or spp., Lagomorpha) with different coat colours. Mamm. Biol. 2007; 72, 224–239.

[22] Melo-Ferreira J, Boursot F, Randi E, Kryukov A, Suchentrunk F, Ferrand N. et al The rise and fall of the mountain hare (*Lepus timidus*) during Pleistocene glaciations: expansion and retreat with hybridization in the Iberian Peninsula. Mol. Ecol. 2007; 16, 605–618. doi: 10.1111/j.1365-294X.2006.03166.x 17257116 10.1111/j.1365-294X.2006.03166.x

[23] Alves P C, Melo-Ferreira J, Freitas H, Boursot P. The ubiquitous mountain hare mitochondria: multiple introgressive hybridization in hares, genus *Lepus*. Phil. Trans. R. Soc. B 2008a; 363, 2831–2839f. doi: 10.1098/rstb.2008.0053 18508749 10.1098/rstb.2008.0053 PMC2606744

[24] Alves PC, Melo-Ferreira J, Branco M, Suchentrunk F, Ferrand N, Harris DJ Evidence for genetic similarity of two allopatric European hares (*Lepus corsicanus* and *L*. *castroviejoi*) inferred from nuclear DNA sequences. Mol. Phylogent. Evol. 2008b; 46, 1191–1197. 10.1016/j.ympev.2007.11.010 18178109

[25] Pietri C, Alves PC, Melo-Ferreira J. Hares in Corsica: high prevalence of *Lepus corsicanus* and hybridization with introduced *L*. *europaeus* and *L*. *granatensis*. Eur. J. Wildl. Res. 2010; 57, 313–321.

[26] Zachos F, Ben Slimen H, Hackländer K, Giacometti M, Suchentrunk F. Regional genetic in situ differentiation despite phylogenetic heterogeneity in Alpine mountain hares. J. Zool. 2010; 282, 47–53.

[27] Wu Y, Xia L, Zhang Q, Yang Q, Meng X. Bidirectional introgressive hybridization between *Lepus capensis* and *Lepus yarkandensis*. Mol. Phylogenet. Evol. 2011; 59, 545–555. doi: 10.1016/j.ympev.2011.03.027 21463697 10.1016/j.ympev.2011.03.027

[28] Melo-Ferreira J, Boursot P, Carneiro M, Esteves PJ, Farelo L, Alves PC. Recurrent introgression of mitochondrial DNA among hares (*Lepus* spp.) revealed by species–tree inference and coalescent simulations. Syst. Biol. 2012; 61, 367–381. doi: 10.1093/sysbio/syr114 22201159 10.1093/sysbio/syr114

[29] Mengoni C, Mucci N, Randi E. Genetic diversity and no evidence of recent hybridization in the endemic Italian hare (*Lepus corsicanus*). Conserv. Genet. 2015; 16, 477–489.

[30] Waltari E, Cook JA Hares on ice: phylogeography and historical demographics of *Lepus arcticus*, *L*. *othus*, and *L*. *timidus* (Mammalia: Lagomorpha). Mol. Ecol. 2005; 14, 3005–3016. doi: 10.1111/j.1365-294X.2005.02625.x 16101770 10.1111/j.1365-294X.2005.02625.x

[31] Ben Slimen H, Suchentrunk F, Ben Ammar Elgaaied A. On shortcomings of using mtDNA sequence divergence for the systematics of hares (genus *Lepus*): An example from cape hares. Mamm. Biol. 2008a;73, 25–32.

[32] Bonhomme F, Fernandez J, Palacios F, Catalan J, Machordon A. Charactérisation biochimique de l'espèce Lepus complexe du genre en Espagne. Mammalia. 1986; 50, 495–506.

[33] Pierpaoli M, Riga F, Trocchi V, Randi E. Species distinction and evolutionary relationships of the Italian hares (*Lepus corsicanus*) as described by mitochondrial DNA sequencing. Mol. Ecol. 1999; 8, 1805–1817. 10620225 10.1046/j.1365-294x.1999.00766.x

[34] Riga F, Trocchi V, Randi E, Toso S.. Morphometric differentiation between the Italian hare (*Lepus corsicanus* De Winton, 1998) and the European brown hare (*Lepus europaeus* Pallas, 1778). J. Zool. 2001; 253, 241–252.

[35] Yom-Tov Y.. On the taxonomic status of the hares (genus *Lepus*) in Israel. Mammalia 1967; 31, 246–259.

[36] Suchentrunk F, Alkon PU, Willing R, Yom-Tov Y. Epigenetic dental variability of Israel hares (*Lepus* sp.): ecogenetic or phylogenetic causation? J. Zool. Lond. 2000; 252, 503–515.

[37] Ben Slimen H, Suchentrunk F, Stamatis C, Mamuris Z, Sert H, Alves PC. et al Population genetics of cape hares (*Lepus capensis* and *L*. *europeaus*): A test for Petter´s hypothesis of conspecificity. Biochem. Syst. Ecol. 2008b; 36, 22–39.

[38] Ben Slimen H, Suchentrunk F, Memmi A, Ben Ammar Elgaaied A. Biochemical genetic relationships among Tunisian hares (*Lepus* sp.) South African hares (*L*. *capensis*), and European brown hare (*L*. *europaeus*). Biochem. Genet. 2005; 43, 577–596. doi: 10.1007/s10528-005-9115-6 16382363 10.1007/s10528-005-9115-6

[39] Palacios F, Angelone C, Alonso G, Reig S.. Morphological evidence of species differentiation within *Lepus capensis* Linnaeus, 1758 (*Leporidae*, *Lagomorph*) in Cape Province, South Africa. Mamm. biol. 2008; 73, 358–370.

[40] Suchentrunk F, Ben Slimen H, Kryger U. Molecular evidence of conspecificity of South African hares conventionally considered *Lepus capensis* L., 1758. Mamm. Biol. 2009; 74, 325–343.

[41] Suchentrunk F, Ben Slimen H, Kryger U. Erratum to ‘‘Molecular evidence of conspecificity of South African hares conventionally considered *Lepus capensis L*., 1758” [Mammalian Biol. 2009; 74 (2009) 325–343]

[42] Scandura M, Iacolina L, Ben Slimen H, Suchentrunk F, Apollonio M.. Mitochondrial CR-1 variation in Sardinian hares and its relationships with other Old World hares (Genus *Lepus*). Biochem. Genet. 2007; 45, 305–312. doi: 10.1007/s10528-007-9076-z 17333330 10.1007/s10528-007-9076-z

[43] Canu A, Suchentrunk F, Cossu A, Foddai R, Iacolina L, Ben Slimen H. et al Differentiation under isolation and genetic structure of Sardinian hares as revealed by craniometric analysis, mitochondrial DNA and microsatellites. J. Zool. Syst. Evol. 2012; 50, 328–337.

[44] Liu J, Chen P, Yu L, Wu SF, Zhang YP, Jiang XL The taxonomic status of *Lepus melainus* (Lagomorpha: Leporidae) based on nuclear DNA and morphological analyses. 2011a; Zootaxa 3010, 47–57.

[45] Nunome M, Kinoshita G, Tomozawa M, Torii H, Matsuki R, Yamada F. et al Lack of association between winter coat colour and genetic population structure in the Japanese hare, *Lepus brachyurus* (Lagomorpha: Leporidae). Biol. J. Linn. Soc. 2014; 11, 761–776.

[46] Petter F. Eléments d´une révision des lièvres Africaines du sous-genre *Lepus*. Mammalia 1959; 23, 41–67.

[47] Angermann R, Feiler A. Zur Nomenklatur, Artabgrenzung und Variabilität der Hasen (Gattung *Lepus*) im westlichen Afrika (*Mammalia*, *Lagomorpha*, *Leporidae*). Zool. Abh. Staatl. Mus. Tierk. Dresden 1988; 43, 149–167.

[48] Smith AT, Johnston CH. Lepus capensis; Lepus fagani; Lepus habessinicus; Lepus microtis; Lepus starcki; Lepus saxatilis. The IUCN Red List of threatened species. Vers. 2014 3, www.iucnredlist.org.2008

[49] Happold DCD. Family LEPORIDAE, hares, rock-hares and rabbits. Leporidae Fischer, 1817. Mém. Soc. Imp. Nat. Moscow, 5: 372, in: Happold D.C.D. (Ed.), Mammals of Africa III. Rodents, Hares and Rabbits, Bloomsbury Publ., London, 2013; pp. 693–717.

[50] Collins L. Supercohort Euarchontaglires, cohort Glires, order Lagomorpha, VII. family Leporidae G. Fischer, 1817. Hares, rabbits, and rock rabbits, in: Skinner JT, Chimimba CT (Eds.), The mammals of the Southern African Subregion, Cambridge Univ. Press, Cambridge, U.K. etc., 2005; pp. 63–76.

[51] Robinson TJ, Dippenaar NJ. Morphometrics of the South African *Leporidae*. II: *Lepus Linnaeus*, 1758, and *Bunolagus* Thomas, 1929. Ann. Transvaal Mus. 1987; 34, 379–404.

[52] Ben Slimen H, Suchentrunk F, Memmi A, Sert H, Kryger U, Alves PC, Ben Ammar Elgaaied A. Evolutionary relationships among hares from North Africa (*Lepus* sp. or *Lepus* spp.), cape hares (*L*. *capensis*) from South Africa, and brown hares (*L*. *europaeus*), as inferred from mtDNA PCR-RLFP and allozyme data. J. Zool. Syst. Evol. 2006; 44, 88–99.

[53] Smith S, Turbill C, Suchentrunk F. Introducing mother’s curse: low male fertility associated with an imported mtDNA haplotype in a captivity colony of brown hares. Mol. Ecol.2010;19, 36–43 doi: 10.1111/j.1365-294X.2009.04444.x 19943893 10.1111/j.1365-294X.2009.04444.x

[54] Andersson AC, Thulin CG, Tegelström H.. Applicability of rabbit microsatellite primers for studies of hybridization between an introduced and native hare species. Hereditas 1999; 130, 309–315. 10509140 10.1111/j.1601-5223.1999.00309.x

[55] Kryger U. Genetic variation among South African hares (Lepus spec.) as inferred from mitochondrial DNA and microsatellites. PhD Thesis, University of Pretoria, Pretoria, South Africa. 2002.

[56] Thulin CG, Stone J, Tegelström H, Walker CW Species assignment and hybrid identification among Scandinavian hares. Wild. Biol. 2006b; 12, 29–38.

[57] Azzaroli-Puccetti ML. The Systematic Relationship of Hares (genus *Lepus*) of the horn of Africa. Cimbebasia 1987a; 9, 1–22.

[58] Azzaroli-Puccetti ML. On the hares of Ethiopia and Somalia and the systematic position of *Lepus whytei* Thomas, 1984 (*Mammalia*, *Lagomrpha*). Atti Accad. Lincei 1987b; 19, 1–19.

[59] Petter F. Nouveaux eléments d’une révision des lièvres Africaines. Mammalia 1963; 27, 238–255.

[60] Yalden D W, Largen M J, Kock D.. Catalogue of the Mammals of Ethiopia. 6. Perissodactyla, Proboscidea, Hyracoidea, Lagomorpha, Tubulidentata, Sirenia and Cetacea. Mon. Zool. Ital., N.S., Suppl. XXI, 1986; 4, 31–103

[61] Yalden D W, Largen M J, Dock D, Hilman JC. Catalogue of the Mammals of Ethiopia and Eritrea. 7. Revised check list, Zoogeography and conservation. Trop. Zool. 1996; 9, 73–164.

[62] Robinson TJ.. Key to South African Leporidae (Mammalia: Lagomorpha). S. Afr. Zool. 1982; 17, 220–222.

[63] Sert H, Suchentrunk F, Erdoğan A. Genetic diversity within Anatolian brown hares (*Lepus europaeus* Pallas, 1778) and differentiation among Anatolian and European populations. Mamm. Biol. 2005; 70, 171–186.

[64] Sert H, Ben Slimen H, Erdoğan A, Suchentrunk F.Mitochondrial HVI sequence variation in Anatolian hares (*Lepus europaeus* Pallas, 1778). Mamm. Biol. 2009; 74, 286–297.

[65] Stamatis C, Giammouli S, Suchentrunk F, Sert H, Stathopoulos C, Mamuris Z,. Recruitment of mitochondrial tRNA gene as auxiliary variability markers for both intra- and inter-species analysis: The paradigm of brown hare (*Lepus europaeus*). Gene 2008; 410, 154–164. doi: 10.1016/j.gene.2007.12.010 18249075 10.1016/j.gene.2007.12.010

[66] Arnason U, Adegoke JA, Bodin K, Born EW, Esa YB, Gullberg A. et al Mammalian mitogenomic relationships and the root of the eutherian tree. Proc. Nat. Acad. Sci. USA 2002;99, 8151–8156. doi: 10.1073/pnas.102164299 12034869 10.1073/pnas.102164299 PMC123036

[67] Werle E, Schneider C, Renner M, Völker M, Fiehn W. Convenient single-step, one tube purification of PCR products for direct sequencing. Nucl. Acid Res. 1994; 22, 4354–4355. 10.1093/nar/22.20.4354 PMC331970 7937169

[68] Hall TA. BioEdit: a user-friendly biological sequence alignment editor program for windows 95/98/NT. Nucl. Acids Symp. Ser. 1999; 41, 95–98.

[69] Stephens M, Smith N, Donnelly P. A New Statistical Method for Haplotype Reconstruction from Population Data. Am. J. Hum. Gent. 2001; 68, 978–989. 10.1086/319501 PMC1275651 11254454

[70] Stephens M, Donnelly P. A Comparison of Bayesian Methods for Haplotype Reconstruction from Population Genotype Data. Am. J. Hum. Gent. 2003; 73, 1162–1169. 10.1086/379378 PMC1180495 14574645

[71] Labrado P, Rozas J.. DNAsp v5. A software for comprehensive analysis of DNA polymorphism data. Bioinformatics 2009; 25, 1451–1452. doi: 10.1093/bioinformatics/btp187 19346325 10.1093/bioinformatics/btp187

[72] Flot JF, TillierA, SamadiS, TillierS. Phase determination from direct sequencing of length-variable DNA regions. Mol. Ecol. 2006; 6, 627–630.

[73] Garick RC, Sunnucks P, Dyer RJ. Nuclear Gene Phylogeography using PHASE: dealing with unresolved genotypes, lost alleles, and systematic bias in parameter estimation. BMC Evol. Biol. 2010; 10, 18 20429950 10.1186/1471-2148-10-118 PMC2880299

[74] Bandelt HJ, Forster P, Röhl A. Median–joining networks for inferring intraspecific phylogenies. Mol. Biol. Evol. 1999; 16, 37–48. 10331250 10.1093/oxfordjournals.molbev.a026036

[75] Hall BG.. Phylogenetic trees made easy A how-to do manual. 4th ed., Sinauer Assoc., Inc. Publishers Sunderland, Mass. U.S.A., 282 pp.2011

[76] Morrison DA. An introduction to phylogenetic networks. RJR Productions, Uppsala, Sweden, 216pp.2011.

[77] Kumar S, Tamura K, Nei M. MEGA: Molecular Evolutionary Genetics Analysis. Pennsylvania State, University, University Park, PA 1993

[78] Tamura K, Peterson D, Peterson N, Stecher G, Nei M, Kumar S. MEGA 5: Molecular Evolutionary Genetics Analysis using Maximum Likelihood, Evolutionary Distance, and Maximum Parsimony Methods. Mol. Biol. Evol. 2011; 28, 2731–2739. doi: 10.1093/molbev/msr121 21546353 10.1093/molbev/msr121 PMC3203626

[79] Saitou N, Nei M. The neighbor-joining methods. A new method for reconstructing phylogenetic trees. Mol. Biol. Evol. 1987; 4, 406–425 3447015 10.1093/oxfordjournals.molbev.a040454

[80] Tamura K, Nei M, Kumar S.. Prospects for inferring very large phylogenies by using the neighobor–joining method. Proc. Nat. Acad. Sci. 2004; 101, 11030–11035. doi: 10.1073/pnas.0404206101 15258291 10.1073/pnas.0404206101 PMC491989

[81] Kimura M. A simple method for estimating evolutionary rate of base substitution through comparative studies of nucleotide sequences. Mol. Evol. 1980; 16, 111–120. 10.1007/BF01731581 7463489

[82] Nei M, Kumar S. Molecular Evolution and Phylogenetics, Oxford University Press, New York 333pp.2000

[83] Posada D.. Selecting models of evolution, in: Lemey P., Salemi M., Vandamme A.-M. (Eds.), The Phylogenetic Handbook. A practical approach to phylogenetic analysis and hypothesis testing. 2nd ed., 5^th^ printing. Cambridge University Press, Cambridge, U.K., 2012; pp. 345–354

[84] Ronquist F, Huelsenbeck J, Teslenco M. Draft MrBayes v.3.2 Manual: Tutorial and Model Summaries. http://mrbayes.sourceforge.net/; 2011

[85] Huelsenbeck J P, BollbackJ P. Empirical and hierarchical Bayesian estimation of ancestral states. Syst. Biol. 2001; 50, 351–366. 12116580

[86] Leache A D, Reeder T W. Molecular systematics of the eastern Fence lizard (*Scelopurus undulatus*): a comparison of parsimony, likelihood, and Bayesian approaches. Syst. Biol. 2002; 51, 44–68. doi: 10.1080/106351502753475871 11943092 10.1080/106351502753475871

[87] FigTree v. 1.4.2. Tree Figure Drawing Tool, A. Rambaut, 2006–2012, Inst. of Evolutionary Biology, Univ. of Edinburgh, 2012; http://tree.bio.ed.ac.uk/

[88] Rico C, Rice I, Webb N, Smith S, Bell D, Hewitt G. Four polymorphic loci for the European wild rabbit, *Oryctolagus cuniculus*. Anim. Genet. 1994; 25, 397. 10.1111/j.1365-2052.1994.tb00379.x 7818179

[89] Surrigudge AK, Bell DJ, Rico C, Hewitt GM. Polymorphic microsatellite loci in the European rabbit (*Oryctolagus cuniculus*) are also in amplified in other Lagomorpha species. Anim. Genet. 1997; 28, 302–305. 9345727 10.1111/j.1365-2052.1997.00137.x

[90] Mougel F, Mounolou JC, Monnerot M. Nine polymorphic microsatellite loci in the rabbit, *Oryctolagus cuniculus*. Animal Genetics. 1997; 28, 58–71. 9124711 10.1111/j.1365-2052.1997.00047.x

[91] Chantry-Darmon C, Urien C, Hayes H, Bertaud M, Chadi-Taourit S, Chardon P, Rogel-Gaillard C. Construction of cytogenetically anchored microsatellite map in rabbit. Mammal. Gen. 2005; 16, 442–459. 10.1007/s00335-005-2471-z 16075371

[92] Van Oosterhout C, Hutchinson WF, Wills DPM, Shipley P. MICRO-CHECKER (Version 2.2.3): Software for identifying and correcting genotyping errors in microsatellite data. Mol. Ecol. 2004; 4, 535–538.

[93] Belhhir K. GENETIX v.4.0 logiciel sous WindowsTM pour la genetique des populations Laboratorio Genome, Populations, Interaction CNRS UMR 5000, Universite de Montpellier II, Montpellier, France, 2004

[94] Raymond M, Rousset F.. GENEPOP (version 1.2): population genetics software for exact tests and ecumenism. J. Hered. 1995; 86, 248–249.

[95] Rice WS. Analyzing tables of statistical tests. Evolution 1989; 43, 223–225. doi: 10.1111/j.1558-5646.1989.tb04220.x 28568501 10.1111/j.1558-5646.1989.tb04220.x

[96] Weir B S, Cockerham CC. Estimating F-statistics for the analysis of population structure. Evol. 1984; 38, 1350–1370. 10.1111/j.1558-5646.1984.tb05657.x 28563791

[97] Garza JC, Freimer NB. Homoplasy for size at microsatellite loci in humans and chaimpanzees. Genome Res. 1996; 6, 211–217. 8963898 10.1101/gr.6.3.211

[98] S-PLUS^®^. Professional Release 2. Lucent Technologies, Inc.; 2000

[99] Cornuet JM, Luikart G. Description and power analysis of two tests for detecting recent population bottlenecks from allele frequency data. Genetics 1996; 144, 2001–2014. 8978083 10.1093/genetics/144.4.2001 PMC1207747

[100] Luikart G, Cornuet JM. Empirical evaluation of a test for identifying recently bottlenecked populations from allele frequency data. Conserv. Biol. 1997; 12, 228–237.

[101] Garza JC, Williamson EG Detection of reduction in population size using data from microsatellite loci. Mol. Ecol. 2001; 10, 305–318. 11298947 10.1046/j.1365-294x.2001.01190.x

[102] Peakall R, Smouse P.E. GenAlEx 6.5: genetic analysis in Excel. Population genetic software for teaching and research—an update. Bioinformatics 2012; 28, 2537–2539. doi: 10.1093/bioinformatics/bts460 22820204 10.1093/bioinformatics/bts460 PMC3463245

[103] Hedrick PW. A standardized genetic differentiation measure. Evolution 2005; 59, 1633–1638. 16329237

[104] Jost L. G_ST_ and its relatives do not measure differentiation. Mol. Ecol. 2008; 17, 4015–4026. 19238703 10.1111/j.1365-294x.2008.03887.x

[105] Dieringer D, Schlötterer C. Microsatellite Analyser (MSA): a platform independent analysis tool for a large microsatellite data set. Mol. Ecol. 2003; 3, 167–169.

[106] Felsenstein J. Phylip (Phylogeny Inference Package), vers. 3.6.9.5. Distrib. by the author, Dept. of Genome Sciences, Univ. of Washington. Seatle, 2013; U.S.A.

[107] Excoffier L, Laval G, Schneider S. Arelequin v.3.11. An integrated software package for population genetics data analysis. Evol. Bioinform. 2005; 1, 47–50. PMC2658868 19325852

[108] Piry S, Alapetite A, Cornuet JM, Paetkau D, Baudouin L, Estoup A. GeneClass2: software for genetic assignment and first generation migrants detection. J. Hered. 2004; 95, 536–539. doi: 10.1093/jhered/esh074 15475402 10.1093/jhered/esh074

[109] Paetkau D, Calvert W, Stirling I, Strobeck C. Microsatellite analysis of population structure in Canadian polar bears. Mol. Ecol. 1995; 4, 347–354. 7663752 10.1111/j.1365-294x.1995.tb00227.x

[110] Paetkau D, Slade R, Burden M, Estoup A. Direct, real-time estimation of migration rate using assignment methods: a simulation-based exploration of accuracy and power. Mol. Ecol. 2004; 13, 55–65. 14653788 10.1046/j.1365-294x.2004.02008.x

[111] Rannala B and Mountain JL Detecting migration by using mutlilocus genotypes. *Proc*. *Nati*. *Acad*. *Sci*. 1997; 94: 9197–9201. 10.1073/pnas.94.17.9197 PMC23111 9256459

[112] Pritchard J K, Stephens M, Donnelly P. Inference of population structure using multilocus genotype data. Genetics 2000; 155, 945–959 10835412 10.1093/genetics/155.2.945 PMC1461096

[113] Falush D, Stephens M, Pritchard JK Inference of Population Structure Using Multilocus Genotype Data: Linked Loci and Correlated Allele Frequencies. Genetics 2003; 164, 1567–1587. 12930761 10.1093/genetics/164.4.1567 PMC1462648

[114] Evano G, Regnaut S, Goudet J. Detecting the number of Clusters of individuals using the software structure: a simulation study. Mol. Ecol. 2005; 14, 2611–2620. doi: 10.1111/j.1365-294X.2005.02553.x 15969739 10.1111/j.1365-294X.2005.02553.x

[115] Earl D A, vonHoldt B M STRUCTURE HARVESTER: a website and program for visualizing STRUCTURE output and implementing the Evano method. Conserv. Genet. Resources 2012; 4, 359–361.

[116] Beerli P, Felsenstein J Maximum likelihood estimation of a migration matrix and effective population sizes in subpopulations by using a coalescent approach. Proc. Nat. Acad. Sci. 2001; 98, 4563–4568. doi: 10.1073/pnas.081068098 11287657 10.1073/pnas.081068098 PMC31874

[117] Flux JEC, Flux MM. Taxonomy and distribution of East African hares. Acta Zool. Fennica. 1983; 174, 41–43.

[118] Chapuis MP, Estoup A Microsatellite Null Alleles and Estimation of Population Differentiation. Mol. Biol. Evol. 2007; 24, 621–631. doi: 10.1093/molbev/msl191 17150975 10.1093/molbev/msl191

[119] Thulin CG, Tegelström H Biased geographical distribution of mitochondrial DNA that passed the species barrier from mountain hares to brown hares (genus Lepus): an effect of genetic incompatibility and mating behavior? J. Zool. 2002; 258, 299–306.

[120] Queller DC, Goodnight F Estimating relatedness using genetic markers. Evolution 1989; 43, 258–275. doi: 10.1111/j.1558-5646.1989.tb04226.x 28568555 10.1111/j.1558-5646.1989.tb04226.x

[121] Belkhir K, Castric V, Bonhomme F. IDENTIX, a software to test for relatedness in a population using permutation methods. Mol. Ecol. 2002; 2, 611–614.

[122] Koutsogiannouli E, Moutou KA, Stamatis C, Mamuris Z. Analysis of MC1R genetic variation in *Lepus* species in Mediterranean refugia. Mamm. Biol. 2012; 77, 428–433.

[123] Ge D, Lissovsky AA, Xia L, Cheng C, Smith AT, Yang Q. Re evaluation of several taxa of Chinese lagomorphs (Mammalia: Lagomorpha) described on the basis of pelage phenotype variation. Mamm. Biol. 2011; 77, 113–123.

[124] Degnan JH, Rosenberg NA. Discordance of species trees with their most likely gene trees. Plos Genet. 2006; 2, 0762–0768. 10.1371/journal.pgen.0020068 PMC1464820 16733550

[125] Matthee CA, Van Vuuren BJ, Bell D, Robinson TJ. A molecular suppermatrix of the rabbits and hares (Leporidae) allows for the identification of five intercontinental exchanges during the Miocene. Syst. Biol. 2004; 53, 433–447. 15503672 10.1080/10635150490445715

[126] Lopes-Martinez N. The Lagomorph fossil record and the origin of the European Rabbit, in: Alves P.C., Ferrand N., Hackländer K. (Eds.), Lagomorph Biology: Evolution, Ecology, and Conservation, Springer, Berlin, Heidelberg; 2008pp. 27–46.

[127] Robinson TJ, Matthee CA Phylogeny and evolutionary origin of the Leporidae: a review of cytogenetics, molecular analyses, and a suppermatrix analysis. Mamm. Rev. 2005; 35, 231–247.

[128] Winkler AJ, Avery DM. Chapter 18: Lagomorpha, in Werdelin L, Sanders WJ, Cenozoic Mammals of Africa. Univ. Calif. Press, Berkely, pp. 2010; 305–317.

[129] Campos J, Goüy de Bellocq J, Schaschl H, Suchentrunk F. MHC class II DQA gene variation across cohorts of brown hares (*Lepus europaeus*) from eastern Austria: testing for different selection hypotheses. Mamm. Biol. 2011; 76, 251–257.

[130] Halanych K M, Demboski J R, Van Vuuren B J, Klein D R, Cook J A. Cytochrome b Phylogeny of North American Hares and Jackrabbits (*Lepus*, *Lagomorpha*) and the Effect of Saturation in Outgroup Taxca. Mol. Phylogent. Evol. 1999; 11, 213–221. 10.1006/mpev.1998.0581 10191066

[131] Robinson TJ, Elder FFB, Chapman JA Karyotype conservatism in the genus *Lepus* (order Lagomorpha). Can. J. Genet. Cytol. 1983; 25, 540–544. 6652568 10.1139/g83-081

[132] Robinson TJ, Yang F, Harrison WR Chromosome painting refines the history of genome evolution in hares and rabbits (order Lagomorpha). Cytogenet. Genome Res. 2002; 96, 223–227. doi: 63034 12438803 10.1159/000063034

[133] Azzaroli-Puccetti ML, Corti M, Scanzani A, Civitelli M V, Capanna E. Karyotypes of two endemic species of hares from Ethiopia, *Lepus habessinicus* and *L*.*starcki* (Lagomorpha, Leporidae). A comparison with *L*. *europaeus*. Mammalia 1996; 60, 223–230.

[134] Wright S. Evolution in Mendelian populations. Genetics 1931; 16, 97–159. 17246615 10.1093/genetics/16.2.97 PMC1201091

[135] Awadi A, Suchentrunk F, Makni M, Ben Slimen H. Variation of partial transferrin sequences and phylogenetic relationships among hares (Lepus capensis, Lagomorpha) from Tunisia. Genetica 2016; doi: 10.1007/s10709-016-9916-z 27485731 10.1007/s10709-016-9916-z

[136] Coyne JA, Orr HA. Speciation. Sinauer Assoc., Inc Mass, 2004; 545 pp.

