# Supplementary figures and images for: Mitochondrial and nuclear DNA reveals reticulate evolution in hares (Lepus spp., Lagomorpha, Mammalia) from Ethiopia
Source: PLoS One. 2017 Aug 2;12(8):e0180137. doi: 10.1371/journal.pone.0180137 (PMC5540492; doi:10.1371/journal.pone.0180137)

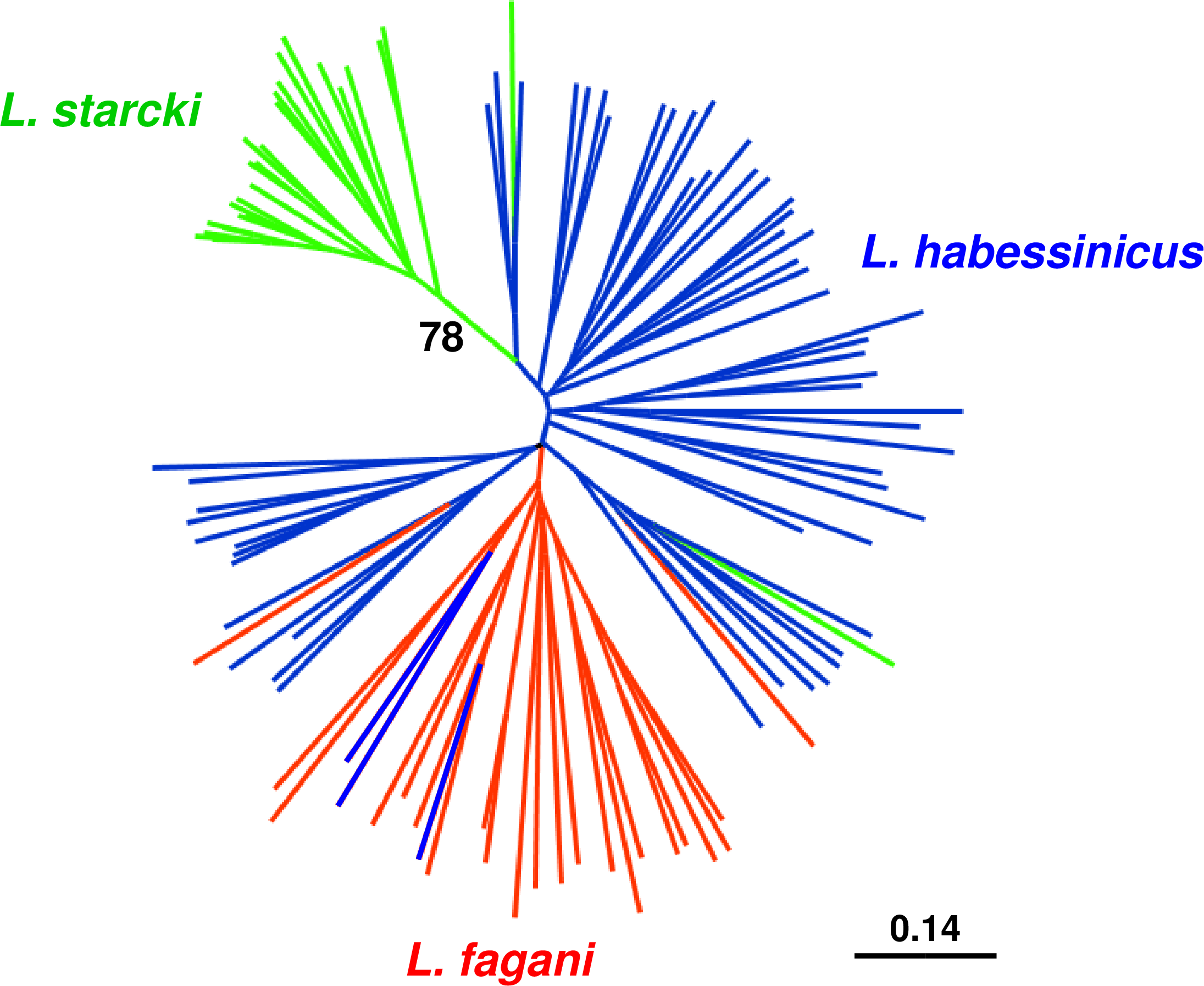

Supplement: S1 Fig — Bootstrap support is shown, if above 50%. For details see “Material and methods”. (TIF) [file pone.0180137.s001.tif]
