# Supplementary material for: Mitochondrial and nuclear DNA reveals reticulate evolution in hares (Lepus spp., Lagomorpha, Mammalia) from Ethiopia
Source: PLoS One. 2017 Aug 2;12(8):e0180137. doi: 10.1371/journal.pone.0180137 (PMC5540492; doi:10.1371/journal.pone.0180137)
Supplement: S1 Table — (DOC) [file pone.0180137.s002.doc]

S1 Table. Accession numbers of the downloaded transferrrin (TF; n = 111; i.e. from NCBI) sequences

| HM233548 | HM233547 | | HM233546 | | HM233545 | | JN037077 | | JN037076 | | JN037075 | | JN037073 | | JN037072.1 | | JN037071 | |
| --- | --- | --- | --- | --- | --- | --- | --- | --- | --- | --- | --- | --- | --- | --- | --- | --- | --- | --- |
| JN037070 | JN037069 | | JN037068. | | JN037067 | | JN037066 | | JN037065 | | JN037064 | | JN037063 | | JN037062 | | JN037061 | |
| JN037060 | JN037059 | | JN037058 | | JN037057 | | JN037056 | | JN037055 | | JN037054 | | HM233558 | | HM233557 | | HM233556 | |
| HM233555 | HM233554 | | HM233553 | | HM233552 | | HM233551 | | HM233550 | | HM233549 | | HM233544 | | HM233543 | | HM233542 | |
| HM233541 | HM233540 | | HM233539 | | HM233538 | | HM233537 | | HM233536 | | HM233535 | | HM233534 | | FJ811637 | | FJ811636 | |
| FJ811635 | FJ811633 | | FJ811632 | | FJ811631 | | FJ811630 | | FJ811626 | | FJ811627 | | FJ811628 | | FJ811629 | | FJ811621 | |
| FJ811622 | FJ811623 | | FJ811624 | | FJ811625 | | FJ811615 | | FJ811616 | | FJ811617 | | FJ811618 | | FJ811619 | | FJ811620 | |
| FJ811634 | FJ811614 | | EU196169 | | EU196168 | | EU196167 | | EU196166 | | EU196165 | | EU196164 | | EU196163 | | EU196162 | |
| EU196161 | AY176279 | | AY176278 | | AY176276 | | AY176277 | | AY176275 | | AY176274 | | AY176273 | | AY176272 | | AY176271 | |
| AY176270 | AY176268 | | AY176267 | | AY176266 | | AY176265 | | AY176264 | | AY176263 | | AY176262 | | AY176261 | | AY176260 | |
| AY176259 | AY176257 | | AY176256 | | AY176254 | | AY176253 | | AY176252 | | AY176251 | | AY176269 | | JN037079 | | JN037078 | |
| AY176280 |  |  |  |  |  |  |  |  |  |  |  |  |  |  |  |  |  |  |

Submitted by Alves et al. [8] and Liu *et al*., unpublished
