# Supplementary material for: Mitochondrial and nuclear DNA reveals reticulate evolution in hares (Lepus spp., Lagomorpha, Mammalia) from Ethiopia
Source: PLoS One. 2017 Aug 2;12(8):e0180137. doi: 10.1371/journal.pone.0180137 (PMC5540492; doi:10.1371/journal.pone.0180137)
Supplement: S2 Table — (DOC) [file pone.0180137.s003.doc]

**S2 Table**. Accession numbers, frequencies, and taxon names of the ATP6 sequences produced in this study; haplotype numbers per taxon are given in parentheses.

| Accession Numbers |  | Frequency | Taxon |
| --- | --- | --- | --- |
| xxxx |  | 2 | cs (2) |
| xxxx |  | 1 | cs (1) |
| xxxx |  | 4 | cs (4) |
| xxxx |  | 1 | cs (1) |
| xxxx |  | 1 | cs (1) |
| xxxx |  | 1 | cs (1) |
| xxxx |  | 5 | cn (5) |
| xxxx |  | 11 | e (8), cn (1), x (2) |
| xxxx |  | 1 | e (1) |
| xxxx |  | 1 | e (1) |
| xxxx |  | 1 | e (1) |
| xxxx |  | 1 | e (1) |
| xxxx |  | 1 | e (1) |
| xxxx |  | 1 | e (1) |
| xxxx |  | 1 | f (1) |
| xxxx |  | 1 | f (1) |
| xxxx |  | 1 | f (1) |
| xxxx |  | 17 | f (2), h (14), x (1) |
| xxxx |  | 1 | f (1) |
| xxxx |  | 1 | f (1) |
| xxxx |  | 1 | f (1) |
| xxxx |  | 3 | f (1), h (2) |
| xxxx |  | 2 | f (1), h (1) |
| xxxx |  | 1 | f (1) |
| xxxx |  | 1 | f (1) |
| xxxx |  | 1 | f (1) |
| xxxx |  | 1 | h (1) |
| xxxx |  | 1 | h (1) |
| xxxx |  | 1 | h (1) |
| xxxx |  | 10 | h (10) |
| xxxx |  | 1 | h (1) |
| xxxx |  | 2 | h (2) |
| xxxx |  | 2 | h (2) |
| xxxx |  | 11 | h (11) |
| xxxx |  | 1 | h (1) |
| xxxx |  | 2 | h (2) |
| xxxx |  | 1 | h (1) |
| xxxx |  | 2 | h (2) |
| xxxx |  | 1 | h (1) |
| xxxx |  | 1 | h (1) |
| xxxx |  | 2 | h (2) |
| xxxx  (S2 Table continued) |  | 1 | h (1) |
| Xxxx |  | 1 | h (1) |
| xxxx |  | 2 | h (2) |
| xxxx |  | 22 | h (1), s (21) |
| xxxx |  | 1 | h (1) |
| xxxx |  | 1 | h (1) |
| xxxx |  | 1 | *Lepus* indet. (from Assosa, Ethiopia) |
| xxxx |  | 1 | x (1) |
| xxxx |  | 1 | x (1) |
| xxxx |  | 1 | s (1) |
| xxxx |  | 1 | s (1) |
| xxxx |  | 3 | s (3) |
| xxxx |  | 1 | s (1) |
| xxxx |  | 1 | cs (1) |
| xxxx |  | 2 | cs (2) |
| xxxx |  | 1 | s (1) |
| xxxx |  | 1 | cs (1) |
| xxxx |  | 2 | t (2) |
| xxxx |  | 1 | cn (1) |
| xxxx |  | 3 | cn (3) |
| xxxx |  | 1 | cn (1) |
| xxxx |  | 1 | cn (1) |
| xxxx |  | 1 | cn (1) |

cs – *L. capensis*, South Africa, cn – *L. capensis*, North Africa, e – *L. europaeus*, f – *L. fagani*, h – *L. habessinicus*, x – *L. saxatilis*, s – *L. starcki*, t – *L. timidus*.
