# Supplementary material for: Mitochondrial and nuclear DNA reveals reticulate evolution in hares (Lepus spp., Lagomorpha, Mammalia) from Ethiopia
Source: PLoS One. 2017 Aug 2;12(8):e0180137. doi: 10.1371/journal.pone.0180137 (PMC5540492; doi:10.1371/journal.pone.0180137)
Supplement: S3 Table — (DOC) [file pone.0180137.s004.doc]

**S3 Table**. Net mean between group (i.e., species) p distances of ATP6 sequences.

|  |  | 1 | 2 | 3 | 4 | 5 | 6 | 7 | 8 |
| --- | --- | --- | --- | --- | --- | --- | --- | --- | --- |
| 1 | CS |  |  |  |  |  |  |  |  |
| 2 | CN | 0.098 |  |  |  |  |  |  |  |
| 3 | E | 0.100 | 0.090 |  |  |  |  |  |  |
| 4 | F | 0.106 | 0.098 | 0.024 |  |  |  |  |  |
| 5 | H | 0.106 | 0.098 | 0.032 | 0.030 |  |  |  |  |
| 6 | X | 0.099 | 0.091 | 0.018 | 0.026 | 0.032 |  |  |  |
| 7 | S | 0.113 | 0.101 | 0.042 | 0.036 | 0.029 | 0.036 |  |  |
| 8 | T | 0.140 | 0.125 | 0.115 | 0.119 | 0.122 | 0.119 | 0.131 |  |

CS – *Lepus capensis*, RSA/South Africa, CN – *L*. *capensis*, North Africa, E – *L. europaeus*, F – *L. fagani*, H – *L. habessinicus*, X – *L. saxatilis*, S – *L. starcki*, T – *L. timidus* (Alps). Minimum and maximum are given in red, respectively.
