# Supplementary material for: Mitochondrial and nuclear DNA reveals reticulate evolution in hares (Lepus spp., Lagomorpha, Mammalia) from Ethiopia
Source: PLoS One. 2017 Aug 2;12(8):e0180137. doi: 10.1371/journal.pone.0180137 (PMC5540492; doi:10.1371/journal.pone.0180137)
Supplement: S4 Table — (DOC) [file pone.0180137.s005.doc]

**S4 Table**. Accession numbers, frequencies, and taxon names of the phased TF sequences produced in this study; haplotype numbers per taxon are given in parentheses.

| Accession  Number |  | Frequency | Taxon |
| --- | --- | --- | --- |
| xxxx |  | 1 | t |
| xxxx |  | 4 | s |
| xxxx |  | 1 | cn |
| xxxx |  | 37 | h (18), s (17), cn (2) |
| xxxx |  | 1 | cn |
| xxxx |  | 4 | cs |
| xxxx |  | 1 | cs |
| xxxx |  | 3 | Oc |
| xxxx |  | 4 | cn |
| xxxx |  | 1 | cn |
| xxxx |  | 1 | cn |
| xxxx |  | 3 | cn |
| xxxx |  | 8 | e |
| xxxx |  | 4 | e |
| xxxx |  | 3 | e |
| xxxx |  | 1 | e |
| xxxx |  | 1 | e |
| xxxx |  | 1 | e |
| xxxx |  | 5 | f |
| xxxx |  | 2 | f |
| xxxx |  | 5 | f |
| xxxx |  | 67 | f (10), h (41), cs (1), x (15) |
| xxxx |  | 5 | f |
| xxxx |  | 9 | f (8), h (1) |
| xxxx |  | 1 | f |
| xxxx |  | 1 | f |
| xxxx |  | 2 | f |
| xxxx |  | 1 | f |
| xxxx |  | 2 | f |
| xxxx |  | 28 | h (21), s (7) |
| xxxx |  | 11 | h |
| xxxx |  | 6 | h |
| xxxx |  | 2 | h |
| xxxx |  | 1 | h |
| xxxx |  | 4 | Oc |
| xxxx |  | 1 | x |
| xxxx |  | 1 | cs |
| xxxx |  | 1 | cs |
| xxxx |  | 1 | cs |
| xxxx |  | 1 | t |
| xxxx |  | 1 | t |
| xxxx |  | 1 | cn |
| xxxx |  | 2 | cn |
| (S4 Table continued)  xxxx |  | 1 | cn |
| xxxx |  | 1 | cn |
| xxxx |  | 1 | cn |
| xxxx |  | 2 | cn |
| xxxx |  | 1 | cn |

cs – *L. capensis*, South Africa, cn – *L. capensis*, North Africa, e – *L. europaeus*, f – *L. fagani*, h – *L. habessinicus*, x – *L. saxatilis*, s – *L. starcki*, t – *L. timidus*, Oc – *Oryctolagus cuniculus*.
