# Supplementary material for: Mitochondrial and nuclear DNA reveals reticulate evolution in hares (Lepus spp., Lagomorpha, Mammalia) from Ethiopia
Source: PLoS One. 2017 Aug 2;12(8):e0180137. doi: 10.1371/journal.pone.0180137 (PMC5540492; doi:10.1371/journal.pone.0180137)
Supplement: S5 Table — CS–L. capensis, South Africa, CN–L. capensis, North Africa, E–L. europaeus, F–L. fagani, H–L. habessinicus, X–L. saxatilis, S–L. starcki, T–L. timidus, M–L. mandshuricus, TW–L. townsendii, CF–L. californicus, AM–L. americanus, OT–L. othus, AR–L. arcticus, CC–L. capensis, China, C–L. corsicanus, G–L. granatensis, OI–L. oiostolus, CO–L. comus, Y–L. yarkandensis, HN–L. hainanus, SI–L. sinensis, CJ–L. castroviejoi. Minimum and maximum are in red, respectively. (DOC) [file pone.0180137.s006.doc]

**S5 Table** Pairwise p-distances of Transferrin sequences. CS – *L. capensis*, South Africa, CN – *L. capensis*, North Africa, E – *L. europaeus*, F – *L. fagani*, H – *L. habessinicus*, X – *L. saxatilis*, S – *L. starcki*, T – *L. timidus*, M – *L. mandshuricus*, TW – *L. townsendii*, CF – *L. californicus*, AM – *L. americanus*, OT – *L. othus* , AR – *L. arcticus*, CC – *L. capensis*, China, C – *L. corsicanus*, G – *L. granatensis*, OI – *L. oiostolus*, CO – *L. comus*, Y – *L. yarkandensis*, HN – *L. hainanus*, SI – *L. sinensis*, CJ – *L. castroviejoi.* Minimum and maximum are in red, respectively.

|  |  | 1 | 2 | 3 | 4 | 5 | 6 | 7 | 8 | 9 | 10 | 11 | 12 | 13 | 14 | 15 | 16 | 17 | 18 | 19 | 20 | 21 | 22 | 23 |
| --- | --- | --- | --- | --- | --- | --- | --- | --- | --- | --- | --- | --- | --- | --- | --- | --- | --- | --- | --- | --- | --- | --- | --- | --- |
| 1 | CS |  |  |  |  |  |  |  |  |  |  |  |  |  |  |  |  |  |  |  |  |  |  |  |
| 2 | CN | 0.013 |  |  |  |  |  |  |  |  |  |  |  |  |  |  |  |  |  |  |  |  |  |  |
| 3 | E | 0.034 | 0.031 |  |  |  |  |  |  |  |  |  |  |  |  |  |  |  |  |  |  |  |  |  |
| 4 | F | 0.015 | 0.012 | 0.032 |  |  |  |  |  |  |  |  |  |  |  |  |  |  |  |  |  |  |  |  |
| 5 | H | 0.012 | 0.010 | 0.030 | 0.005 |  |  |  |  |  |  |  |  |  |  |  |  |  |  |  |  |  |  |  |
| 6 | X | 0.012 | 0.010 | 0.030 | 0.003 | 0.003 |  |  |  |  |  |  |  |  |  |  |  |  |  |  |  |  |  |  |
| 7 | S | 0.013 | 0.010 | 0.024 | 0.010 | 0.007 | 0.008 |  |  |  |  |  |  |  |  |  |  |  |  |  |  |  |  |  |
| 8 | T | 0.033 | 0.030 | 0.009 | 0.031 | 0.029 | 0.030 | 0.024 |  |  |  |  |  |  |  |  |  |  |  |  |  |  |  |  |
| 9 | M | 0.014 | 0.010 | 0.030 | 0.012 | 0.010 | 0.010 | 0.010 | 0.029 |  |  |  |  |  |  |  |  |  |  |  |  |  |  |  |
| 10 | TW | 0.030 | 0.027 | 0.004 | 0.028 | 0.026 | 0.026 | 0.020 | 0.005 | 0.026 |  |  |  |  |  |  |  |  |  |  |  |  |  |  |
| 11 | CF | 0.025 | 0.022 | 0.025 | 0.024 | 0.021 | 0.022 | 0.019 | 0.026 | 0.022 | 0.022 |  |  |  |  |  |  |  |  |  |  |  |  |  |
| 12 | AM | 0.030 | 0.027 | 0.013 | 0.029 | 0.026 | 0.027 | 0.022 | 0.014 | 0.027 | 0.009 | 0.022 |  |  |  |  |  |  |  |  |  |  |  |  |
| 13 | OT | 0.034 | 0.031 | 0.008 | 0.032 | 0.030 | 0.031 | 0.024 | 0.007 | 0.030 | 0.004 | 0.026 | 0.013 |  |  |  |  |  |  |  |  |  |  |  |
| 14 | AR | 0.028 | 0.025 | 0.013 | 0.026 | 0.024 | 0.025 | 0.020 | 0.011 | 0.024 | 0.009 | 0.024 | 0.016 | 0.011 |  |  |  |  |  |  |  |  |  |  |
| 15 | CC | 0.012 | 0.008 | 0.029 | 0.010 | 0.008 | 0.008 | 0.008 | 0.028 | 0.009 | 0.025 | 0.020 | 0.025 | 0.029 | 0.023 |  |  |  |  |  |  |  |  |  |
| 16 | C | 0.035 | 0.031 | 0.009 | 0.033 | 0.031 | 0.031 | 0.025 | 0.010 | 0.031 | 0.005 | 0.026 | 0.014 | 0.009 | 0.014 | 0.030 |  |  |  |  |  |  |  |  |
| 17 | G | 0.032 | 0.032 | 0.010 | 0.034 | 0.032 | 0.032 | 0.026 | 0.011 | 0.031 | 0.006 | 0.027 | 0.015 | 0.010 | 0.014 | 0.030 | 0.011 |  |  |  |  |  |  |  |
| 18 | OI | 0.023 | 0.019 | 0.021 | 0.021 | 0.019 | 0.019 | 0.017 | 0.021 | 0.019 | 0.017 | 0.025 | 0.019 | 0.021 | 0.020 | 0.018 | 0.022 | 0.023 |  |  |  |  |  |  |
| 19 | CO | 0.028 | 0.025 | 0.014 | 0.026 | 0.024 | 0.025 | 0.020 | 0.015 | 0.024 | 0.010 | 0.026 | 0.016 | 0.014 | 0.016 | 0.023 | 0.013 | 0.016 | 0.017 |  |  |  |  |  |
| 20 | Y | 0.014 | 0.011 | 0.024 | 0.013 | 0.011 | 0.011 | 0.010 | 0.024 | 0.010 | 0.020 | 0.021 | 0.022 | 0.025 | 0.020 | 0.009 | 0.025 | 0.025 | 0.017 | 0.021 |  |  |  |  |
| 21 | HN | 0.013 | 0.010 | 0.021 | 0.012 | 0.009 | 0.010 | 0.008 | 0.021 | 0.010 | 0.017 | 0.012 | 0.017 | 0.021 | 0.017 | 0.008 | 0.022 | 0.022 | 0.013 | 0.017 | 0.011 |  |  |  |
| 22 | SI | 0.013 | 0.010 | 0.030 | 0.012 | 0.009 | 0.010 | 0.010 | 0.030 | 0.010 | 0.026 | 0.022 | 0.027 | 0.031 | 0.025 | 0.007 | 0.031 | 0.032 | 0.019 | 0.025 | 0.011 | 0.010 |  |  |
| 23 | CJ | 0.035 | 0.031 | 0.009 | 0.033 | 0.031 | 0.031 | 0.025 | 0.010 | 0.030 | 0.005 | 0.026 | 0.014 | 0.009 | 0.014 | 0.030 | 0.000 | 0.011 | 0.021 | 0.012 | 0.025 | 0.021 | 0.031 |  |
